# Lonidamine, a Novel Modulator for the BvgAS System of *Bordetella* Species

**DOI:** 10.1111/1348-0421.13193

**Published:** 2024-12-15

**Authors:** Natsuko Ota, Takashi Nishida, Daron M. Standley, Aalaa Alrahman Sherif, Satoshi Iwano, Dendi Krisna Nugraha, Toshiya Ueno, Yasuhiko Horiguchi

**Affiliations:** ^1^ Department of Molecular Bacteriology, Research Institute for Microbial Diseases Osaka University Suita Japan; ^2^ Department of Genome Informatics, Research Institute for Microbial Diseases Osaka University Suita Japan; ^3^ Immunology Frontier Research Center Osaka University Suita Japan; ^4^ Center for Infectious Disease Education and Research Osaka University Suita Japan; ^5^ Institute for Tenure Track Promotion University of Miyazaki Miyazaki Japan

**Keywords:** *Bordetella*, BvgAS, lonidamine, modulator, phase conversion

## Abstract

The Gram‐negative bacteria *Bordetella pertussis*, *B. parapertussis*, and *B. bronchiseptica* cause respiratory diseases in various mammals. They share the BvgAS two‐component system, which regulates the phenotypic conversion between the virulent Bvg^+^ and avirulent Bvg^–^ phases. In the BvgAS system, the sensor kinase BvgS senses environmental cues and transduces a phosphorelay signal to the response regulator BvgA, which leads to the expression of Bvg^+^ phase‐specific genes, including virulence factor genes. Bacteria grown at 37°C exhibit the Bvg^+^ phenotype. In contrast, at lower than 26°C or in the presence of modulators, such as MgSO_4_ and nicotinic acid, the BvgAS system is inactivated, leading bacteria to the avirulent Bvg^–^ phase. Therefore, effective modulators are expected to provide a therapeutic measure for *Bordetella* infection; however, no such modulators are currently available, and the mechanism by which modulators inactivate the BvgAS system is poorly understood. In the present study, we identified lonidamine as a novel modulator after screening an FDA‐approved drug library using bacterial reporter systems with the Bvg^+^‐specific and Bvg^–^‐specific promoters. Lonidamine directly bound to the VFT2 domain of BvgS and inactivated the BvgAS system at concentrations as low as 50 nM, which was at least 2000‐ to 20,000‐fold lower than the effective concentrations of known modulators. Lonidamine significantly reduced the adherence of *B. pertussis* to cultured cells but unexpectedly exacerbated bacterial colonization of the mouse nasal septum. These results provide insights into the structural requirements for BvgAS modulators and the role of Bvg phenotypes in the establishment of infection.

**Abbreviations**
Ak: Akaluc
ANOVA: a one‐way analysis of variance
BG: Bordet−Gengou
CDC: Centers for Disease Control and Prevention
DMSO: dimethyl sulfoxide
DPBS: Dulbecco's modified phosphate‐buffered saline
GB1: B1 immunoglobulin‐binding domain of streptococcal protein G
gDNA: genomic DNA
HBSS: Hank's balanced salt solution
ITC: isothermal titration calorimetry
LCA: low‐casamino acids
Lon: lonidamine
M: mock
mC: mCherry
qPCR: quantitative PCR
RFU: relative fluorescence units
RLU: relative luminescence units
SS: Staine−Scholte
Thm: Tohama
*vag*s: virulence‐activated genes
*vrg*s: virulence‐repressed genes
Wt: wild type

## 1 Introduction

The Gram‐negative bacteria, *Bordetella pertussis*, *B. parapertussis*, and *B. bronchiseptica*, which are collectively called classical *Bordetella*, cause respiratory diseases in various mammals [1, 2]. *B. pertussis* and *B. parapertussis* are the causative agents of the human disease pertussis or whooping cough. *B. bronchiseptica* infects various mammals, including immunocompromised humans [1, 3]. These bacterial species are genetically related: genome analyses indicate that *B. pertussis* and *B. parapertussis* independently evolved from *B. bronchiseptica* or a *B. bronchiseptica*‐like ancestor [1, 4, 5]. Pertussis caused by *B. pertussis* and *B. parapertussis* is characterized by severe coughing. The bacteria disseminate into the lungs and cause necrotizing bronchiolitis, intra‐alveolar hemorrhage, and fibrinous edema [1, 6, 7]. In severe cases, extreme lymphocytosis occurs, occasionally leading to intractable pulmonary hypertension, respiratory failure, and death [1, 6]. Pertussis is preventable by vaccination, and vaccination coverage is currently more than 80% worldwide [8]. However, the disease has shown a resurgence since 2000, with a higher incidence in adolescents, hypothetically because of waning immunity raised by acellular vaccines and/or antigenic changes in bacteria [7, 9, 10, 11]. In addition, macrolide‐resistant *B. pertussis* is being increasingly isolated in Asia, Europe, and North America [12, 13, 14, 15, 16, 17]. Macrolides are used as the first‐line antibiotics for the treatment of pertussis. More than 90% of clinical isolates in China were found to be resistant to macrolides [18, 19, 20]. The Centers for Disease Control and Prevention (CDC) reported the emergence of antibiotic‐resistant *B. pertussis* as a potential threat, which is based on the monitoring of resistance in the United States by testing isolates received through the CDC's Emerging Infections Program (CDC's 2019 AR Threats Report). These circumstances have prompted the development of therapeutic approaches without antibiotics to control pertussis.

Classical *Bordetella* species share many virulence factors, and their pathogenicity is commonly regulated by the BvgAS two‐component system, which consists of the sensor kinase BvgS and response regulator BvgA [1]. At approximately 37°C, the optimal growth temperature and mammalian body temperature, BvgS transduces a phosphorelay signal to BvgA. BvgA, which functions as a transcription regulator, subsequently activates the expression of virulence‐activated genes (*vag*s), including toxins (adenylate cyclase toxin, dermonecrotic toxin, and pertussis toxin [specific to *B. pertussis*]), adhesins (filamentous hemagglutinin and fimbriae), effectors of the type III secretion system, and autotransporters (pertactin and Vag8) [21, 22]. Simultaneously, BvgA indirectly represses a cluster of genes called virulence‐repressed genes (*vrg*s). This virulent phenotypic state is called the Bvg^+^ phase, in which bacteria infect hosts [23]. In contrast, the BvgAS system is inactivated at ambient temperatures (lower than 26°C) or in the presence of modulators for BvgS, such as nicotinic acid and MgSO_4_, and bacteria lose infectivity, repressing the expression of *vag*s [23, 24]. In this avirulent phenotype, called the Bvg^–^ phase, bacteria express *vrg*s, some of which are involved in metabolic pathways [25], and adapt to survive in the extra‐host environments. The mechanism through which lower temperatures lead to the inactivation of the BvgAS is unknown. In contrast, modulators are considered to inactivate the BvgAS system by directly interacting with BvgS [26, 27]; however, the mode of interaction is poorly understood. Natural modulating ligands for BvgS also remain unknown. If we can obtain an effective modulator, it may be a valuable tool to dissect the mechanism by which BvgS is inactivated. In addition, it may provide a therapeutic measure without antibiotics for pertussis and other *Bordetella* infections. Nicotinic acid and MgSO_4_, known modulators, of which effective concentrations are as high as 5 and 20 mM, respectively [28, 29, 30], are difficult to use for such purposes.

In this study, in order to find novel effective modulators, we constructed several reporter strains of *B. pertussis* with Bvg^+^‐ and Bvg^–^‐specific promoters to monitor Bvg states, screened an FDA‐approved drug library, and identified lonidamine, an antineoplastic agent [31], as a modulator, which efficiently inactivates the BvgAS system at 50 nM by directly binding to BvgS. The mode of interaction between lonidamine and BvgS was examined through a docking simulation. We also investigated whether lonidamine controls *B. pertussis* infection by leading the bacteria to the Bvg^–^ phase at the cultured cell and animal levels. Treatment with lonidamine inhibited bacterial adherence to cultured cells. However, unexpectedly, it exacerbated bacterial colonization in mice.

## 2 Materials and Methods

### 2.1 Bacterial Strains and Culture Conditions

The bacterial strains used in this study are listed in Table S1. *B. pertussis* strains Tohama and 18323 were maintained in our laboratory. *B. pertussis* clinical strains BP140 and BP142 were provided by K. Kamachi (National Institute of Infectious Diseases). *B. bronchiseptica* RB50 [24] was provided by P. A. Cotter (University of California). *B. parapertussis* 12822 was provided by Kitasato University through M. Watanabe (currently Nihon Pharmaceutical University). *Bordetella* strains were grown on Bordet‐Gengou agar (Becton Dickinson, 248200) containing 1% Hypolypeptone (SHIOTANI M.S., 390‐02116), 1% glycerol, 10 µg/mL ceftibuten, and 15% defibrinated horse blood (BG) or 10 mg/mL BSA (Wako, 015‐27053) instead of defibrinated horse blood (BG‐BSA). The bacteria recovered from colonies on BG plates were suspended in Stainer−Scholte (SS) medium [32] to an optical density at 650 nm (OD_650_) of 0.2 for *B. pertussis* and *B. parapertussis* and 0.02 for *B. bronchiseptica*, and were incubated with shaking at 37°C overnight. *B. pertussis* BP140 and BP142 were grown from 0.2 OD_650_ with shaking at 37°C in low‐casamino acids (LCA) medium [33] overnight. Unless otherwise specified, the resultant overnight culture was used as starting bacterial samples. *B. pertussis* in the Bvg^–^ phase was obtained by cultivation in the presence of 50 mM MgSO_4_. Colony‐forming units (CFU) were estimated from OD_650_ values according to the following equation: 1 OD_650_ = 3.3 × 10^9^ CFU/mL. *E. coli* was grown on Luria−Bertani agar or medium. Growth media were supplemented with antibiotics when necessary at the following concentrations: ampicillin, 100 µg/mL; gentamicin, 10 µg/mL; and kanamycin, 25 µg/mL.

### 2.2 Reagents

Lonidamine (Tokyo Chemical Industry, L0283) was dissolved in dimethyl sulfoxide (DMSO) at 500 mM and kept as a stock solution. Fludarabine (Tokyo Chemical Industry, F0658) and otilonium bromide (Wako, QC‐4672) were dissolved at 2 mM in DMSO. Dydrogesterone (Tokyo Chemical Industry, P2623), methyl 1‐(2,4‐dichlorobenzyl)‐1H‐indazole‐3‐carboxylic acid (Aaron Chemicals, AR002RL6), adjudin (MedChemExpress, HY‐18996), and indazole‐3‐carboxylic acid (Tokyo Chemical Industry, I0672) were dissolved at 20 mM in DMSO. An FDA‐approved drug library of 1134 compounds was provided by the Compound Library Screening Center, Osaka University. Akalumine‐HCl (Wako, 016‐26704) was dissolved in 0.9% NaCl at 60 mM as a stock solution.

### 2.3 Construction of Plasmids and Mutants

Mutant strains derived from *B. pertussis* Tohama were constructed by double‐crossover homologous recombination between *sacB*‐based suicide vectors and bacterial genomes as previously described with slight modifications [34]. The desired mutant strains with genomic integration were selected by cultivation in SS medium containing 10% sucrose. The plasmids and primers used in this study are listed in Tables S2 and S3, respectively. The desired mutants were confirmed by DNA sequencing. Unless otherwise specified, DNA ligation was performed with an In‐Fusion HD cloning kit (TaKaRa Bio, 639649) or In‐Fusion snap assembly master mix (TaKaRa Bio, 638949).

A Bvg^–^ phase‐locked strain carrying a deletion of the 542nd to 1020th amino acid region in BvgS and a Bvg^+^ phase‐locked strain carrying a substitution of His for Arg at the 570th amino acid position in BvgS were constructed from *B. pertussis* Tohama as previously described with slight modifications [24, 34]. A DNA fragment containing the *bvgS* region was amplified by PCR with *B. pertussis* 18323 genomic DNA (gDNA) as a template and a combination of the primers BvgS‐region1Bp‐S+ and BvgS‐region2Bp‐AS+. The resultant fragment was inserted into the *Sma*I site of pABB‐CRS2‐Gm. Inverse PCR was performed with the resultant plasmid and combinations of the primers BvgS‐region2‐S and BvgS‐region1‐AS for the Bvg^–^ phase‐locked mutant and BB2995‐C3mutation‐S and BB2995‐C3mutation‐AS for the Bvg^+^ phase‐locked mutant. The resultant PCR products were phosphorylated with T4 polynucleotide kinase (TaKaRa Bio, 2021) and recircularized with T4 DNA ligase (Promega, M180A). The generated plasmids, pABB‐CRS2‐Gm‐∆BvgS_542−1020_ and pABB‐CRS2‐Gm‐BvgS_R570H_, were introduced into *E. coli* DH5α λ*pir* and transferred into *B. pertussis* by triparental conjugation with helper strain *E. coli* HB101 harboring pRK2013 [35], which was provided by K. Minamisawa (Tohoku University).

Other *bvgS* mutants were constructed as previously described with slight modifications [36]. The *bvgS* gene was amplified by PCR using *B. pertussis* Tohama gDNA and the primers BvgS_full‐F and BvgS_full‐R. The resultant fragment was inserted into linear pABB‐CRS2‐Gm, which was generated by inverse PCR with the primers BD267‐F and BD264‐R. The resultant plasmid was named pABB‐CRS2‐Gm‐Tohama BvgS_full_. The BvgS F375A, R380A, T462A + S465A, and quadA mutants were constructed by site‐direct mutagenesis of the *bvgS* gene through inverse PCR using pABB‐CRS2‐Gm‐Tohama BvgS_full_ and the appropriate primers shown in Table S3. Amplified PCR products were phosphorylated and recircularized using T4 polynucleotide kinase and T4 DNA ligase, respectively. The resultant plasmids were introduced into *E. coli* S17‐1 λ*pir* (R380A) or DH5α λ*pir* (F375A, T462A + S465A, and quadA) and transferred into *B. pertussis* Bvg^–^ phase‐locked strain by biparental conjugation or triparental conjugation with helper strain *E. coli* HB101 harboring pRK2013, respectively. The mutation of the *bvgS* gene in the resultant mutants was confirmed by sequencing.

We used the intergenic region between BP3747 and BP3748 of the *B. pertussis* genome as the site for the genomic integration of foreign genes [37]. To achieve this, the intergenic region was amplified by PCR using the primers Up‐BP3747‐F1 and Down‐BP3748‐R1 and inserted into linear pABB‐CRS2‐Gm, which was generated by inverse PCR with the primers BD267‐F and BD264‐R. The resultant plasmid was designated pABB‐CRS2‐Gm‐Tohama BP3747‐BP3748 and used for the following construction. P_*tac*_‐Akaluc Thm was generated as follows. A DNA fragment covering the *Akaluc opt* region was amplified by PCR from pEX_K4J2_Akaluc^opt^ (Eurofins Genomics K.K.), in which codons of the *Akaluc* gene are optimized for expression in *B. bronchiseptica*, with the primers Akaluc opt S+ and Akaluc opt AS+. The amplified fragment was inserted into the *Eco*RI site of pBBR1MCS5‐P_*tac*_, a derivative of pBBR1MCS5‐P_*tac*_‐*gfp* [34]. The resultant plasmid carrying a *tac* promoter (P_*tac*_), *Akaluc opt* gene, and *trpA* terminator (T_*trpA*_) was named pBBR1MCS5‐P_*tac*_‐Akaluc opt‐T_*trpA*_, from which the P_*tac*_–*Akaluc opt*–T_*trpA*_ region was amplified by PCR using the primers pABB‐Akaluc‐F1 and pABB‐Akaluc‐R1. The amplified fragment was inserted into the BP3747–BP3748 intergenic region of linear pABB‐CRS2‐Gm‐Tohama BP3747−BP3748, which was generated by inverse PCR with the primers Down‐BP3748‐F1 and Up‐BP3747‐R1. The resultant plasmids were introduced into *E. coli* S17‐1 λ*pir* and transconjugated into *B. pertussis* by biparental conjugation. The desired mutants were selected as described above and confirmed by the luminescence expression of colonies grown on BG plates sprayed with 100 µM akalumine. P_*vrgX*_‐Akaluc Thm was generated as follows. A DNA fragment of the promoter region of the *vrgX* gene (P_*vrgX*_) was amplified by PCR using *B. pertussis* Tohama gDNA as the template and the primers In‐fusion BRP1340‐Fw and In‐fusion BRP1340‐Akaluc‐Rv. Another DNA fragment covering the *Akaluc opt* gene was amplified by PCR from pBBR1MCS5‐P_*tac*_‐Akaluc opt‐T_*trpA*_ with the primers Akaluc opt‐Fw and Akaluc opt AS +. The DNA fragments of P_*vrgX*_ and the *Akaluc opt* gene were ligated and inserted into the *Eco*RI*–Bam*HI site of pBBR1MCS5‐P_*tac*_. The resultant plasmid, which carried the P_*vrgX*_–*Akaluc opt*–T_*trpA*_ region, was designated pBBR1MCS5‐BRP1340‐Akaluc opt. The P_*vrgX*_ *–Akaluc opt*–T_*trpA*_ region of the plasmid was amplified by PCR using the primers pABB‐BRP1340‐Akaluc‐F1 and pABB‐BRP1340‐Akaluc‐R1. The PCR product was inserted into linear pABB‐CRS2‐Gm‐Tohama BP3747‐BP3748, which was generated by inverse PCR with the primers Inverse‐BP3748‐F1 and Inverse‐BP3747‐R1. The resultant plasmid was introduced into *E. coli* S17‐1 λ*pir* and transferred into *B. pertussis* by biparental conjugation. To generate mCherry Thm, a DNA fragment covering the P_*tac*_–*mCherry2* region was amplified by PCR with pBBR1MCS5‐TpR‐P_*tac*_‐mCherry2‐T_*trpA*_ (laboratory collection) as the template and the primers pABB‐mCherry‐F1 and pABB‐mCherry‐R1. The PCR product was inserted into linear pABB‐CRS2‐Gm‐Tohama BP3747‐BP3748, which was generated by inverse PCR with the primers Inverse‐BP3748‐F1 and Inverse‐BP3747‐R1. The resultant plasmid was introduced into *E. coli* S17‐1 λ*pir* and transconjugated into *B. pertussis* by biparental conjugation. The desired mutants were selected as described above and confirmed by colony PCR using the primers Ki‐31 and pABB‐check‐R1.

Mutant strains carrying GFP‐reporter plasmids were generated as follows. DNA fragments of the promoter regions of Bvg^+^‐specific genes (P_*fhaB*_, P_*cya*_, P_*dnt*_, P_*prn*_, P_*ptx*_, and P_*vag8*_) and Bvg^–^‐specific genes (P_*vrgX*_, P_*vrg6*_, P_*vrg73*_, P_*bp1618*_, P_*bp1738*_, and P_*kpsM*_) were amplified by PCR using gDNA from *B. pertussis* Tohama or 18323 as the template with the appropriate primers shown in Table S3. The *gfp* gene was amplified by PCR with pBBR1MCS5‐P_*tac*_‐*gfp*. Each DNA fragment of the promoter regions and the *gfp* gene were independently inserted into the *Eco*RI–*Bam*HI site of pBBR1MCS5‐P_*tac*_ or pBBR1MCS5‐P_*tac*_ *‐gfp*. These plasmids were introduced into *E. coli* DH5α λ*pir* and transferred into *B. pertussis* Tohama wild type, *bvgS* mutants, and mCherry Thm by triparental conjugation with helper strain *E. coli* HB101 harboring pRK2013.

### 2.4 Purification of Recombinant Proteins

Recombinant proteins of the VFT1, VFT2, and VFT1–VFT2 (VFT1 + 2) domains of BvgS were generated as previously reported [26] with slight modifications. The recombinant VFT1 and VFT1 + 2 proteins were designed to be tagged with the B1 immunoglobulin‐binding domain of streptococcal protein G (GB1) to increase the solubility of proteins [26, 38]. A DNA fragment ranging from the GB1 gene to hexa‐histidine peptide gene of the GEV2 vector (Addgene, 12616) was amplified by PCR using the primers GEV2‐insert‐F1 and GEV2‐insert‐R1. The resultant fragment was ligated to linear pColdII (TaKaRa Bio, 3362) missing the hexa‐histidine gene, which had been generated by inverse PCR with the primers pCold‐inverse‐F1 and pCold‐inverse‐R1. The resultant plasmid was designated pColdII‐GEV2. DNA fragments covering the VFT1, VFT2, and VFT1 + 2 genes were amplified by PCR using pABB‐CRS2‐Gm‐BvgS_full_ as the template with the primers indicated in Table S3. The resultant DNA fragments, which carry unique restriction sites, *Bam*HI–*Xho*I for VFT1 and VFT1 + 2 and *Bam*HI–*Hin*dIII for VFT2, were introduced into the corresponding sites of pColdII‐GEV2 and pColdII, respectively, using a T4 DNA ligation Kit. The resultant plasmids were designated pColdII‐GB1‐VFT1, pColdII‐GB1‐VFT1 + 2, and pColdII‐VFT2. These plasmids were independently introduced into *E. coli* BL21 (DE3), and VFT1, VFT2, and VFT1 + 2 were expressed in bacteria that were incubated in the presence of 1 mM isopropyl‐β‐D‐thiogalactopyranoside at 15°C for 24 h. Bacteria were then disrupted by sonication in 50 mM sodium phosphate buffer, pH 7.4, containing 300 mM NaCl (Buffer A). Sonicated suspensions were centrifuged at 12,000 × *g* at 4°C for 5 min. The supernatants were mixed with an equal volume of Buffer A containing 15 mM imidazole and independently applied to a column of the HIS‐Select Nickel Affinity Gel (Merck Millipore, P6611) equilibrated with Buffer A containing 7.5 mM imidazole. After non‐absorbed substances were washed out of the column with Buffer A containing 7.5 mM imidazole, recombinant proteins were eluted with Buffer A containing 200 mM imidazole. Recombinant VFT1 and VFT2 for isothermal titration calorimetry (ITC) were purified using the ÄKTA pure 25 M1 system with HisTrap HP (Cytiva) with the same buffer system. The eluted fractions of VFT1 and VFT2 were dialyzed against 50 mM sodium phosphate, pH 7.4. The eluted VFT1 + 2 fractions were dialyzed against Buffer A.

### 2.5 Antibody

Three‐week‐old female Wister rats were intraperitoneally injected with 10 µg of purified VFT1 + 2 emulsified with complete Freund's adjuvant (Becton Dickinson, 263810), followed by two booster injections with 25 µg of the same antigen emulsified with incomplete Freund's adjuvant (Becton Dickinson, 263910) every 2 weeks. Rats were bled 9 days after the last immunization and their sera were obtained.

### 2.6 Immunoblotting

Starting bacterial samples were suspended in SS medium at 0.2 OD_650_ and incubated at 37°C for 24 h. Bacterial pellets, which were collected by centrifugation at 8000 × *g* for 10 min, were resuspended in Dulbecco's modified phosphate‐buffered saline (DPBS) and disrupted by sonication with a Bioruptor (Cosmo Bio). Sonicated suspensions were centrifuged at 12,000 × *g* for 5 min, and cell lysates were obtained after the filtration of supernatants through 0.2 µm pore membranes (Thermo Fisher Scientific). Samples were mixed with a 1/5 volume of 312.5 mM Tris–HCl, pH 6.8, containing 33% glycerol, 10% SDS, and 0.1% bromophenol blue, and subjected to SDS‐PAGE followed by electrotransfer to a polyvinylidene difluoride membrane (Millipore, Immobilon‐P). The membrane was subjected to immunoblotting with the combination of rat anti‐VFT1 + 2 (1:10,000) and goat anti‐rat IgG‐HRP (Santa Cruz, sc‐2032, 1:10,000) antibodies. The target proteins were visualized by enhanced chemiluminescence using an Immobilon Western (Merck Millipore, WBKLS0500), and signals were detected with Amersham Imager 600 UV (GE Healthcare). The membrane was further treated with stripping solution (Nacalai Tesque, 05364‐55), reprobed with the combination of rabbit anti‐FtsZ [39] (1:10,000) and goat anti‐rabbit IgG‐HRP (Jackson, 111‐035‐144, 1:10,000) antibodies, and subjected to chemiluminescence detection as described above.

### 2.7 Quantitative PCR (qPCR)

The starting bacterial samples of *B. pertussis* Tohama, 18323, BP140, and BP142, *B. parapertussis* 12822, and *B. bronchiseptica* RB50 were diluted to 0.1 OD_650_ for Tohama, 18323, and RB50 and 0.2 OD_650_ for BP140, BP142, and 12822 with SS medium containing 1 µM lonidamine or 50 mM MgSO_4_ and were then incubated at 37°C. After OD_650_ values became 0.5 to 1, 500 µL aliquots of the culture were mixed with an equal volume of RNAprotect bacteria reagent (QIAGEN, 76506). Total RNA was extracted using NucleoSpin RNA (TaKaRa Bio, 740955) and treated with recombinant DNase I (TaKaRa Bio, 2270A). cDNA was synthesized from total RNA by RT‐PCR using the PrimeScript RT‐PCR kit (TaKaRa Bio, RR037A) and subjected to qPCR with the StepOnePlus real‐time PCR system (Applied Biosystems) using Fast SYBR Green master mix (Thermo Fisher Scientific, 4385612) and appropriate primers. The relative expression levels of each gene were calculated with the comparative threshold cycle (*C*_t_) method (∆∆*C*_t_) after standardization by the *recA* gene.

### 2.8 GFP Reporter Assay

Four distinct *B. pertussis* reporter strains, Thm/P_*fhaB*_‐*gfp*,/P_*ptx*_‐*gfp*,/P_*vrgX*_‐*gfp*, and/P_*vrg73*_‐*gfp* were used (Table S1). Reporter assays were performed for liquid cultures of SS medium and solid cultures of BG‐BSA agar. In the liquid culture assay, 300 µL of the starting bacterial samples of the reporter strains at 0.1 OD_650_ was further incubated without shaking in the wells of a 96‐well plate at 37°C for 3 days. In the solid culture assay, 200 µL of BG‐BSA agar was solidified in the wells of a 96‐well black plate (Thermo Fisher Scientific, 137101) and allowed to dry for a few hours. Each chemical compound was diluted to the desired concentrations with BG‐BSA solution (0.45% Potato infusion powder (Sigma‐Aldrich, 52424), 0.55% NaCl, 1% Hypolypeptone, 1% glycerol, 10 µg/mL ceftibuten, and 10 mg/mL BSA) and 50 µL of the dilutions or 50 mM MgSO_4_ were allowed to permeate into the agar for 2−3 days. Three microliters of the starting bacterial samples of the reporter strains at 0.5 OD_650_ were poured on the agar and incubated at 37°C for 3 days. The fluorescence intensity of GFP expressed by the bacteria in each well was measured by GLOMAX MULTI with Ex: 490 nm and Em: 510–570 nm or the GLOMAX Discover System with Ex: 475 nm and Em: 500–550 nm (Promega) and expressed as relative fluorescence units (RFU).

### 2.9 ITC

Purified VFT1 and VFT2 preparations were dialyzed against 50 mM sodium phosphate buffer, pH 7.4. The interaction between VFT1/VFT2 and lonidamine was analyzed with MicroCal PEAQ‐ITC Automated (Malvern, Worcestershire, UK) according to the manufacturer's instructions.

### 2.10 In Silico Molecular Docking

The crystal structure of the *B. pertussis* BvgS periplasmic domain (PDB ID: 4Q0C) was docked with lonidamine (Mol2 ID: D07257) using the Schrödinger suite (Schrödinger, Release 2021). The protein structure was prepared using the Protein Preparation Wizard and default settings were applied to other parameters. The Grid box was generated using the Grid Receptor Generation tool centered on residues Leu374 to Ser465. The lonidamine molecule was processed with the LigPrep tool, generating multiple 3D conformations, which were then docked using Glide to chain C of the 4Q0C (dimeric model of BvgS periplasmic domain) protein. Docking scores were analyzed, and the optimal binding pose was visualized using PyMOL (https://www.pymol.org).

### 2.11 In Vitro Adherence Assay

Human lung epithelial A549 cells were maintained in Dulbecco's Modified Eagle's Medium high glucose (Sigma, D5796) supplemented with 10% fetal bovine serum (HyClone; Cytiva, SH30910.03). Cells were seeded at 2 × 10^4^ cells/well in the wells of a 24‐well plate, in which a 13 mm circular cover glass had been placed, and then incubated under a 5% CO_2_ atmosphere at 37°C for 24 h. Cells were washed with 20 mM Hepes‐buffered Hank's balanced salt solution, pH 7.4 (HBSS‐Hepes), three times and infected with bacteria as follows. Starting bacterial samples of mCherry Thm/P_*fhaB*_‐*gfp* and/P_*vrgX*_‐*gfp* were prepared in the presence of 0.5% DMSO or 1 µM lonidamine. After two washes with HBSS‐Hepes by centrifugation, the bacteria were suspended in the same buffer at a concentration of 4 × 10^6^ CFU/mL. Fifty microliters of the bacterial suspension and 450 µL of HBSS‐Hepes were added to the wells of the A549 culture. The cells in the plate were spun at 200 × *g* for 5 min and then incubated under 5% CO_2_ at 37°C for 24 h. After 24 h, the cells were washed with HBSS‐Hepes and fixed with DPBS containing 4% paraformaldehyde. The specimens on the cover glass were stained with Prolong diamond antifade mountant with DAPI (Invitrogen, R36966) and subjected to microscopy on a FV3000 confocal microscope (EVIDENT). Images of a field were captured with different channels for GFP, mCherry, and DAPI. The number of mCherry‐ and GFP‐positive bacteria that attached to the cells was counted using the images processed with ImageJ software (https://imagej.net).

### 2.12 In Vivo Imaging of *B. pertussis*‐Infected Mice

Six‐week‐old male C57BL/6J mice were anesthetized with 2% isoflurane (Wako). The starting bacterial samples of P_*tac*_‐Akaluc Thm and P_*vrgX*_‐Akaluc Thm, which were prepared in the presence or absence of 1 µM lonidamine, were intranasally inoculated into anesthetized mice at 1 × 10^7^ CFU/25 µL/mouse using a micropipette with a needle‐like tip. The stock solution of lonidamine dissolved in DMSO was diluted in 99 mM Tris and 767 mM glycine, pH8.57, as previously reported [40], and intranasally injected into mice at 10 mg/kg body weight every day. On Days 1, 4, and 8 of infection, mice were intraperitoneally injected with 300 µL of akalumine‐HCl in 0.9% NaCl to an injection dose of 50 nmol/g body weight, and bioluminescence images of the Akaluc‐expressing *B. pertussis* strains were immediately acquired with IVIS Lumina LT series III (Revvity, Massachusetts, USA). Image acquisition conditions were as follows: exposure time = 20 min, binning = medium, height = 1.5 cm, and f/stop = 1.2. Luminescence levels in the nasal septum were measured by Living Image 4.7 software.

### 2.13 Others

The protein concentrations of cell lysates were measured using the Micro BCA protein assay kit (Thermo Fisher Scientific, 23235). Luminescence intensity from the Akaluc–Akalumine reaction in bacterial cultures was measured by the GLOMAX Discover System with 350–700 nm and 0.5 s of integration. Statistical analyses were performed using Prism 9 (GraphPad Software). Significance is expressed as follows: *p < 0.05, **p < 0.01, ***p < 0.001, ****p < 0.0001. In all analyses, the significance of differences was defined as *p* < 0.05.

## 3 Results

### 3.1 Suitable Promoters for the GFP Reporter Assay

**Figure 1:**
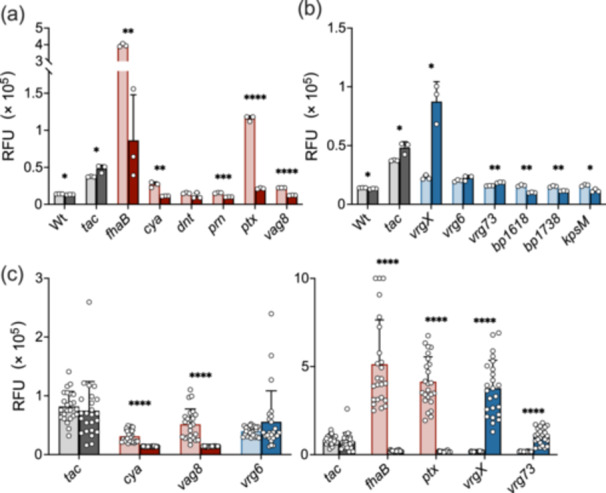
GFP‐expressing *Bordetella pertussis* reporter strains to indicate Bvg states. GFP reporter strains with Bvg^+^ phase‐dependent promoters (*fhaB*, *cya*, *dnt*, *prn*, *ptx*, and *vag8*; red) and Bvg^–^ phase‐dependent promoters (*vrgX*, *vrg6*, *vrg73*, *bp1618*, *bp1738*, and *kpsM*; blue) were incubated in SS medium (a, b) and on BG‐BSA agar (c) in the presence (dark color) or absence (light color) of 50 mM MgSO_4_ for 3 days, and the fluorescence intensity of expressed GFP was measured. The *tac* promoter was used as the BvgAS‐independent control. The wild type (Wt) does not carry the reporter plasmid. Note that the *Y*‐axis scale is different for each panel. Values represent the mean ± SD (*n* = 3 for a and b, and *n* = 24 for c). The significance of differences was analyzed by an unpaired *t*‐test on each row with Holm−Šídák's multiple comparisons test.

To screen for compounds that lead *B. pertussis* to the Bvg^–^ phase by inactivating the BvgAS system, we tried to construct reporter strains in which GFP was properly expressed under regulation by Bvg‐dependent promoters. To achieve this, we generated various *B. pertussis* reporter strains, which carried the GFP gene downstream of the Bvg^+^‐dependent promoters [41, 42, 43, 44], P_*fhaB*_, P_*cya*_, P_*dnt*_, P_*prn*_, P_*ptx*_, and P_*vag8*_, or the Bvg^–^‐dependent promoters [25, 45, 46, 47, 48], P_*vrgX*_, P_*vrg6*_, P_*vrg73*_, P_*bp1618*_, P_*bp1738*_, and P_*kpsM*_. These reporter strains were incubated in SS medium in the presence or absence of 50 mM MgSO_4_, which is known as a modulator of the BvgAS system, and GFP expression was estimated (Figure 1a,b). GFP expression driven by each Bvg^+^‐dependent promoter was reduced by MgSO_4_. Among the Bvg^–^‐dependent promoters, P_*vrgX*_, P_*vrg6*_, and P_*vrg73*_ were activated by MgSO_4_, whereas P_*bp1618*_, P_*bp1738*_, and P_*kpsM*_ were not. Therefore, we subjected P_*fhaB*_, P_*ptx*_, P_*cya*_, and P_*vag8*_, the activity of which was markedly reduced by MgSO_4_, and P_*vrgX*_, P_*vrg73*_, and P_*vrg6*_, the activity of which was stimulated by MgSO_4_, to the following examinations with BG‐BSA agar. A bacterial culture on BG‐BSA agar, which is easier to handle than SS liquid medium, is suitable for the high‐throughput screening of a compound library. Similar to the cultivation in SS medium, all of the selected promoters responded to MgSO_4_. Notably, the reporter strains carrying P_*fhaB*_, P_*ptx*_, P_*vrgX*_, and P_*vrg73*_ were more sensitive than the other reporter strains (Figure 1c, right panel). Based on these results, we decided to use the GFP‐expressing *B. pertussis* reporters with P_*fhaB*_, P_*ptx*_, P_*vrgX*_, and P_*vrg73*_ in screening.

### 3.2 Screening for Chemical Compounds to Convert *B. pertussis* to the Bvg^–^ Phenotype

**Figure 2:**
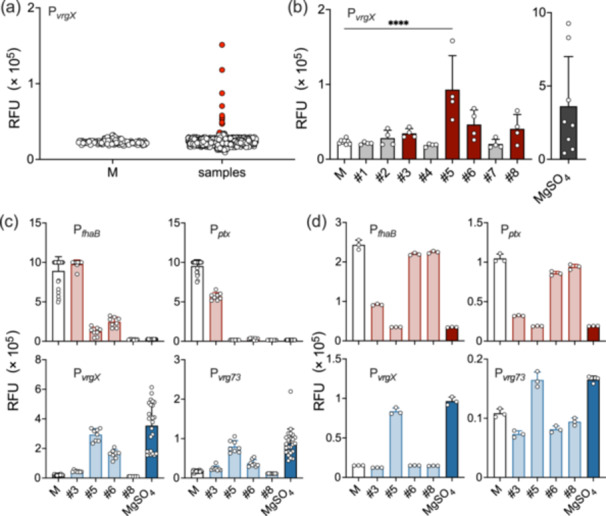
Screening of chemical compounds that inactivate the BvgAS system of *Bordetella pertussis*. (a) An FDA‐approved drug library consisting of 1134 chemical compounds was screened for activity stimulating the expression of GFP using the Thm/P_*vrgX*_‐*gfp* strain. Eleven compounds that increased GFP expression levels by more than 1.5‐fold the average of the negative control (mock, M) are indicated as red spots. (b–d) Candidate compounds after the first screening were further examined using Thm/P_*vrgX*‐_ *gfp* (b–d), Thm/P_*vrg73*‐_ *gfp* (c, d), Thm/P_*fhaB*‐_ *gfp* (c, d), and Thm/P_*ptx*‐_ *gfp* (c, d). Reporter strains were grown on BG‐BSA agar (a–c) or in SS medium (d). Each compound was applied at 10 µM (a and b), 30 µM (c), or under bactericidal concentrations (d; 10 µM [#5, #8], and 30 µM [#3, #6]). Numbered compounds are etoposide (#1), artemisinin (#2), fludarabine (#3), econazole nitrate (#4) lonidamine (#5), dydrogesterone (#6), auranofin (#7), and otilonium bromide (#8). As the control, 50 mM MgSO_4_ was applied (b−d). Values represent the mean ± SD (*n* = 4 for b, *n* = 8 for c, and *n* = 3 for d). Data were statistically analyzed by a one‐way analysis of variance (ANOVA) with Tukey's multiple comparisons test (b).

We initially screened the FDA‐approved drug library consisting of 1134 compounds using the P_*vrgX*_ reporter strain, which enables positive selection. After cultivating reporter bacteria on BG‐BSA agar containing each compound of the library, we found 11 compounds that increased GFP expression level by more than 1.5‐fold the average of the mock control (Figure 2a, red spots). Of the 11 candidates, three compounds were excluded: two appeared to be intrinsically fluorescent and one was nalidixic acid, a quinolone antibiotic. The remaining eight candidates were tentatively numbered and further examined with four replicates for each compound. In these experiments, compounds #3, #5, #6, and #8, but not #1, #2, #4, or #7, increased GFP expression to levels that were 1.5‐ to 4‐fold higher than the mock control (Figure 2b). Therefore, the former compounds were subjected to the following examination using the P_*fhaB*_, P_*ptx*_, P_*vrgX*_, and P_*vrg73*_ reporter strains that were cultivated on BG‐BSA agar and in SS medium.

**Figure 3:**
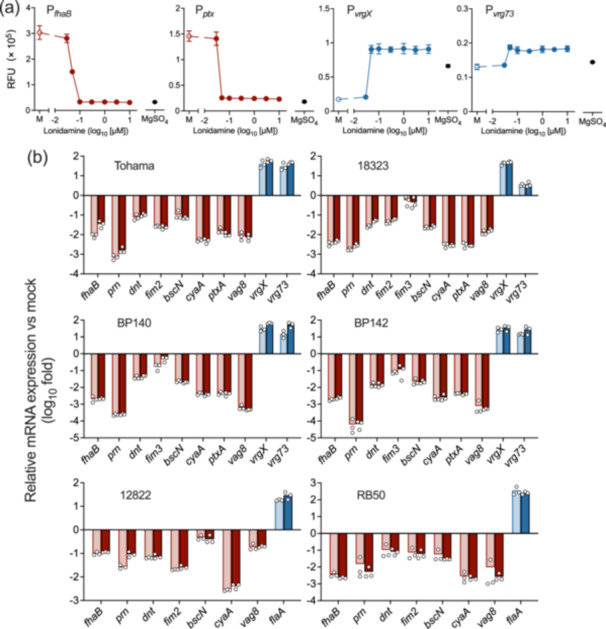
Lonidamine converts classical *Bordetella* species to the Bvg^–^ phase. (a) The GFP reporter assay using Thm/P_*fhaB*‐_ *gfp*, Thm/P_*ptx*‐_ *gfp*, Thm/P_*vrgX*‐_ *gfp*, and Thm/P_*vrg73*‐_ *gfp* strains incubated in SS medium containing 0.03−10 µM lonidamine, 1.5% DMSO (mock, M), or 50 mM MgSO_4_. Representative data are shown after three independent experiments under similar conditions. Values represent the mean ± SD (*n* = 3). Some error bars that are shorter than the symbol size are not depicted. (b) The relative expression levels of Bvg^+^ phase‐specific genes (*fhaB*, *prn*, *dnt*, *fim2*, *fim3*, *bscN*, *cyaA*, *ptxA*, and *vag8*; red) and Bvg^–^ phase‐specific genes (*vrgX*, *vrg73*, and *flaA*; blue) of *B. pertussis* (Tohama, 18323, BP140, and BP142), *B. parapertussis* 12822, and *B. bronchiseptica* RB50. Bacteria were grown in SS medium containing 50 mM MgSO_4_ (dark color) or 1 µM lonidamine (light color) and subjected to qPCR, as described in the Materials and Methods. Data represent fold changes in the expression of each gene compared to bacteria grown in SS medium containing 1.5% DMSO (*n* = 3).

In the reporter assay with BG‐BSA agar (Figure 2c), the effects of compound #3 on P_*fhaB*_ and P_*ptx*_ were negligible. Compound #8 inhibited all the reporter strains from expressing GFP. Compound #8 at the test concentration (30 µM) was considered to be bacteriostatic or bactericidal as described below. Compounds #5 and #6 increased the activity of the Bvg^–^‐dependent promoters, P_*vrgX*_ and P_*vrg73*_, and reduced that of the Bvg^+^‐dependent promoters P_*fhaB*_ and P_*ptx*_, exerting the desired effects. In the assay using SS medium, we initially examined the effects of the compounds on bacterial growth (Supporting Information S1: Figure S1) and adopted the maximal concentrations of each compound that did not reduce or inhibit growth in the Bvg‐dependent promoter assays (Figure 2d). In these experiments, compound #8 at 20 µM completely inhibited bacterial growth. Compound #5 exerted the desired effects, similar to those in the BG‐BSA assay, while compound #6 did not affect any promoter activities. Therefore, we selected compound #5, lonidamine (1‐(2,4‐dichlorobenzyl)‐1H‐indazole‐3‐carboxylic acid), as the most probable candidate. Lonidamine inactivated and activated the Bvg^+^‐ and Bvg^–^‐dependent promoters, respectively, at concentrations as low as 50 nM (Figure 3a). In the qPCR assay, lonidamine exerted similar effects on the expression of various Bvg‐dependent genes of the *B. pertussis* strains, *B. parapertussis*, and *B. bronchiseptica* (Figure 3b). Based on these results, we decided to further characterize lonidamine as a modulator for the BvgAS system.

### 3.3 Lonidamine Interacts With the Periplasmic VFT2 Domain of BvgS

**Figure 4:**
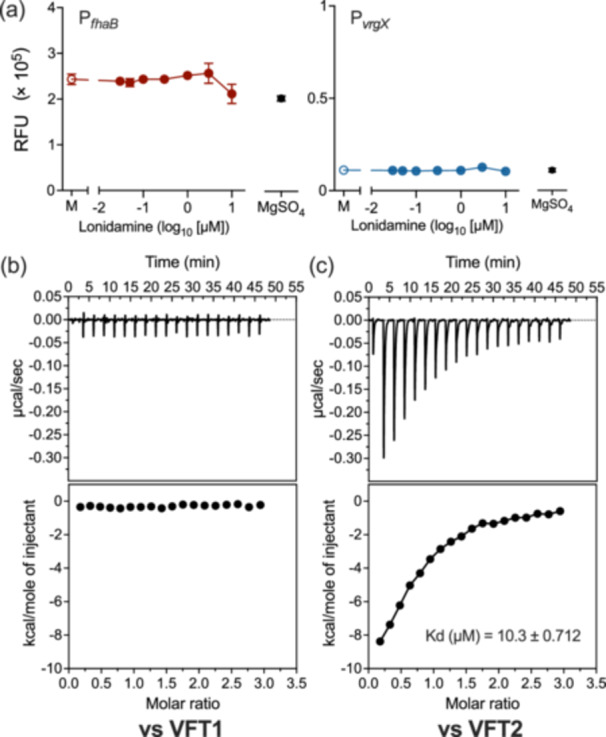
Lonidamine targets BvgS but not BvgA. (a) The GFP reporter assay using the Bvg^+^ phase‐locked mutants of Thm/P_*fhaB*‐_ *gfp* and Thm/P_*vrgX*‐_ *gfp* incubated in SS medium containing 0.03−10 µM lonidamine, 1.5% DMSO (mock, M), or 50 mM MgSO_4_. Data are shown from a single experiment with three independent samples. Values represent the mean ± SD (*n* = 3). Some error bars that are shorter than the symbol size are not depicted. (b, c) The interaction of the VFT domains of BvgS and lonidamine analyzed by ITC. The upper panels show the rate of heat released by 2 µL injections of 300 µM lonidamine into a cell containing 20 µM VFT1 (b) or VFT2 (c). The lower panels show the integrated areas of the respective peaks in the upper panel plotted against the molar ratio of lonidamine to VFT1 (b) or VFT2 (c).

In the BvgAS system, the sensor kinase BvgS transduces signals from extracellular cues to the downstream response regulator BvgA, which activates the expression of Bvg^+^‐dependent genes and indirectly represses Bvg^–^‐dependent genes [49]. Therefore, we examined whether lonidamine interacted with BvgS or BvgA to inactivate the BvgAS system by examining a Bvg^+^ phase‐locked strain (*B. pertussis* BvgS_R570H_), in which the BvgAS system is continuously active due to a mutation in BvgS. The Bvg^+^ phase‐locked strain was insensitive to lonidamine, suggesting that the target site of lonidamine is BvgS but not BvgA (Figure 4a). BvgS comprises two periplasmic domains, VFT1 and VFT2 [26, 50]. ITC revealed that lonidamine bound to VFT2 but not VFT1 (Figure 4b,c, Supporting Information S1: Figure S2a).

**Figure 5:**
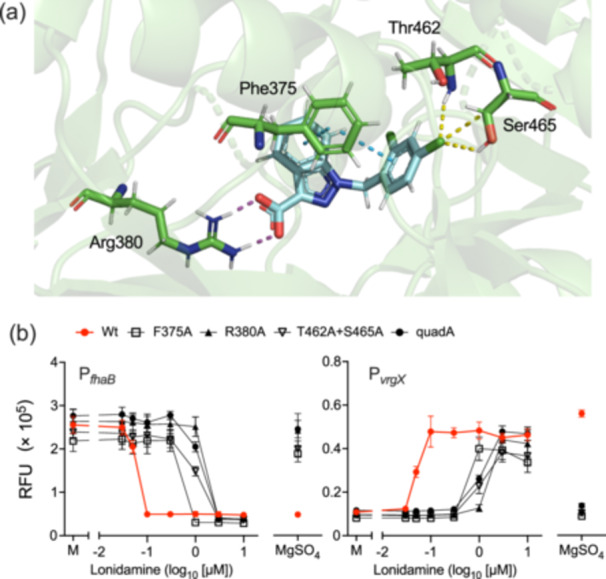
Putative interaction mode of lonidamine with VFT2. (a) Docking simulation of lonidamine (cyan) and VFT2 (green) in the dimeric state. The structure of VFT2 is represented as a ribbon diagram. Lonidamine and the amino acid residues involved in the interaction are shown as sticks. The magenta and yellow dotted lines represent hydrogen and halogen bonds, respectively. The blue dotted lines represent the π–π interaction. The glide score of the model is −7.862. (b) The P_*fhaB*_‐ and P_*vrgX*_‐based reporter assay using *Bordetella pertussis bvgS* mutants (F375A, R380A, T462A + S465A, and quadA). Bacteria were incubated in SS medium containing 0.03−10 µM lonidamine, 1.5% DMSO (mock, M), or 50 mM MgSO_4_, and expressed GFP was estimated as described in the Materials and Methods. Values represent the mean ± SD (*n* = 3).

To understand the mode of interaction between VFT2 and lonidamine, we conducted molecular docking simulations of the BvgS periplasmic region (PDB ID: 4Q0C) [51] using Glide [52]. The simulation predicted lonidamine encased in a cavity of VFT2, which corresponds to the cavity previously indicated as the putative ligand‐binding site [26, 51]. In the cavity, Phe375 interacted with the aromatic rings of lonidamine via a π–π stacking interaction (4.1–4.4 Å), Arg380 with the carboxyl group via a hydrogen bond (< 2.5 Å), and Thr462 and Ser465 with the chlorobenzyl group via a halogen bond (2.5 and 2.9–3.0 Å, respectively) (Figure 5a). We examined *B. pertussis* reporter strains with substitutions of these amino acids in BvgS in the P_*fhaB*_ and P_*vrgX*_ reporter assays (Figure 5b, Supporting Information S1: Figure S2b). These mutant strains were 8‐ to 26‐fold less sensitive to lonidamine than the wild‐type strain (Figure 5b and Table 1), suggesting the involvement of these amino‐acid residues in the interaction.

**Table 1:** EC_50_ of lonidamine on *Bordetella pertussis bvgS* mutants.

Reporter strains	EC_50_ of lonidamine (µM)^a^	
	P_*fhaB*_	P_*vrgX*_
Wild type	0.058	0.050
F375A	0.520	0.404
R380A	1.494	1.217
T462A + S465A	1.054	1.030
quadA	1.188	1.081

^a^ EC_50_ values were calculated from the data shown in Figure 5b using Prism 9.

**Figure 6:**
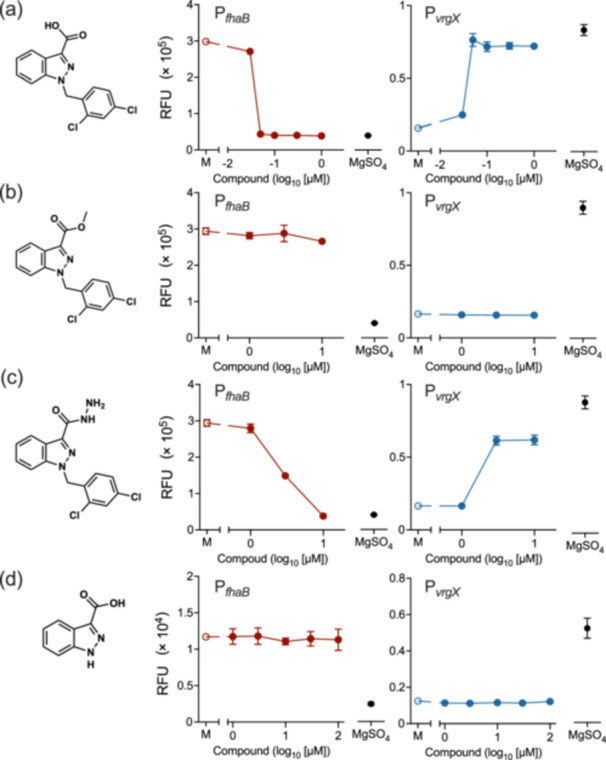
Effects of lonidamine and its analogs on Bvg states of *Bordetella pertussis*. Thm/P_*fhaB*_‐*gfp* and Thm/P_*vrgX*_‐*gfp* were incubated in SS medium containing 1.5% DMSO (mock, M) or the indicated concentrations of lonidamine and its analogs or 50 mM MgSO_4_, and expressed GFP was estimated as described in the Materials and Methods. The chemical structural formulas of lonidamine (1‐(2,4‐dichlorobenzyl)‐1H‐indazole‐3‐carboxylic acid (a), methyl 1‐(2,4‐dichlorobenzyl)‐1H‐indazole‐3‐carboxylic acid (b), adjudin (1‐(2,4‐dichlorobenzyl)‐1H‐indazole‐3‐carbohydrazide) (c), and indazole‐3‐carboxylic acid (d) are shown on the left of each panel. Experiments for (a−c) were performed at least twice and representative data are shown. For (d), data from a single experiment are shown. Values represent the mean ± SD (*n* = 3 for each panel). Some error bars that are shorter than the symbol size are not depicted.

We also examined several lonidamine analogs for their ability to inactivate BvgS (Figure 6). Lonidamine comprises dichlorobenzene and indazole carboxylic acid, which are linked by a carbon‐nitrogen covalent bond. The effects on BvgS of analogs of lonidamine, in which the carboxylic acid is methyl esterified (Figure 6b) or dichlorobenzene is excluded (Figure 6d), were abrogated. The activity of these analogs was not observed at concentrations up to 100 µM (data not shown), indicating the importance of the carboxylic acid and dichlorobenzene moieties of lonidamine for the interaction with VFT2. On the other hand, adjudin, which carries carboxylic acid hydrazide, exhibited an activity that was 200‐fold less than lonidamine (Figure 6c). Considering that the analog with methyl esterified carboxylic acid was inactive (Figure 6b), it can be concluded that the hydrazide group functions to interact with VFT2, but the methyl group does not. However, the details of this difference currently remain unknown.

### 3.4 Effects of Lonidamine on *B. pertussis* Cell Adherence and Mouse Infection

**Figure 7:**
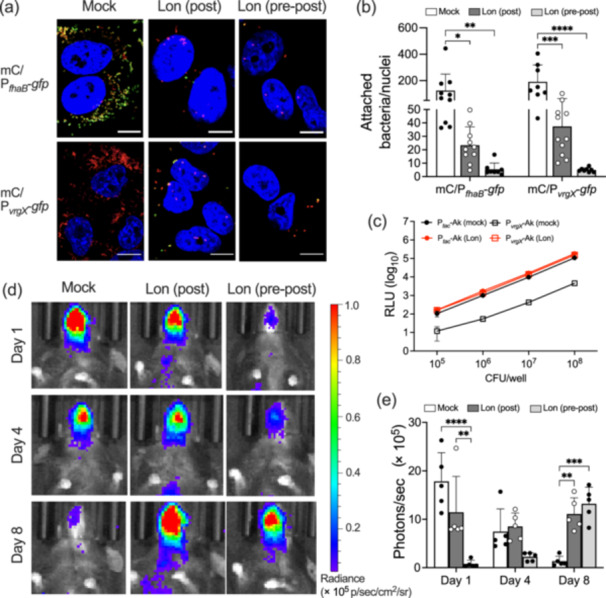
Effects of lonidamine on *Bordetella pertussis* infection. (a) Microscopic images of A549 cells infected with mCherry Thm/P_*fhaB*_‐*gfp* (mC/P_*fhaB*_‐*gfp*, upper panel) or mCherry Thm/P_*vrgX*_‐*gfp* (mC/P_*vrgX*_‐*gfp*, lower panel). Bacteria were precultured with (“pre−post”) or without (“mock” and “post”) 1 µM lonidamine (Lon) and added to a culture of A549 cells as described in the Materials and Methods. A549 cells with bacteria were incubated for 24 h in the presence (“post” and “pre−post”) or absence (“mock”) of 1 µM lonidamine. In the mock control, 0.5% DMSO was applied. In the “post” culture, 1 µM lonidamine was applied after bacteria adhered to cells for the first hour. Bar, 10 µm. (b) Numerical data on mCherry‐positive bacteria adhering to A549 cells. Microscopic images were captured from at least eight independent fields and the number of adhering bacteria was counted. A single plot symbol represents a single microscopic field. (c) The correlation between luminescence intensity and the number of P_*tac*_‐Akaluc Thm (P_*tac*_‐Ak) and P_*vrgX*_‐Akaluc Thm (P_*vrgX*_‐Ak) strains. Serially diluted bacterial samples precultured with or without 1 µM lonidamine (Lon) were mixed with 100 µM akalumine in a 96‐well black plate. The luminescence intensity in each well was measured as described in the Materials and Methods and expressed as relative luminescence units (RLU). (d) Effects of lonidamine on *Bordetella pertussis* infection in mice. P_*tac*_‐Akaluc Thm was precultured with (“pre−post”) or without (“mock” and “post”) 1 µM lonidamine and intranasally inoculated into anesthetized mice at 1 × 10^7^ CFU/25 µL/mouse (Day 0). Mice were intranasally injected with (“post” and “pre−post”) or without (“mock”) 10 mg/kg body weight of lonidamine every day from Day 1. On Days 1, 4, and 8 of infection, mice were intraperitoneally injected with Akalumine‐HCl, and bioluminescence images of bacteria were acquired. On Day 1, images were acquired 7 h before the lonidamine injection. In the mock control, 4.6% DMSO was applied. (e) Quantitative data on luminescence levels in the nasal septum estimated by Living Image 4.7 software. Values represent the mean ± SD (b, c and *n* = 5 for e). The significance of differences was analyzed by a two‐way ANOVA with Šídák's multiple comparisons test (b, e).

The aforementioned results indicate that lonidamine is the most effective modulator to date. *Bordetella* species in the Bvg^–^ phase are known to lose infectivity. Therefore, lonidamine has the potential to be a candidate for controlling *Bordetella* infection by inactivating the BvgAS system. To examine this, we subjected lonidamine to experiments with *B. pertussis* infection at the cellular and animal levels using A549 cells and mice. At the cellular level, we estimated bacterial adherence to A549 cells using mCherry‐expressing *B. pertussis*, which carried the P_*fhaB*_ or P_*vrgX*_‐driven GFP reporter plasmid (Figure 7a,b). Bacteria were treated with lonidamine before (“pre−post”) and after (“post” and “pre−post”) the adherence of bacteria to the cells. In both experimental groups, the number of mCherry‐positive bacteria adhering to A549 cells was lower than that of the mock control. The effects of the “pre−post” treatment with lonidamine were more marked than those of the “post” treatment. In these culture assays, the P_*fhaB*_‐driven GFP expression of bacteria was reduced and P_*vrgX*_‐driven GFP expression was stimulated, confirming that lonidamine actually converted the adhering bacteria to the Bvg^–^ phase (Supporting Information S1: Figure S3a).

In the mouse infection, we utilized the AkaBLI system [53] with Akaluc‐expressing *B. pertussis* to evaluate bacterial colonization by bioluminescence intensity (Figure 7c−e). On Day 1 of infection, bacterial colonization was reduced in the experimental groups treated with lonidamine, similar to the results of experiments at the cellular level. However, unexpectedly, colonization was exacerbated in the lonidamine‐treated groups on Day 8 compared to the mock group. When the P_*vrgX*_‐Akaluc reporter strain of *B. pertussis* was subjected to similar experiments, Akaluc‐dependent luminescence was detected through the infection period in mice treated with lonidamine, indicating that lonidamine actually converted the colonizing bacteria to the Bvg^–^ phase (Supporting Information S1: Figure S3b,c).

## 4 Discussion

In the present study, we found lonidamine as a novel modulator that inactivates the BvgAS system of *Bordetella* species by binding to the VFT2 domain of BvgS. Lonidamine (1‐(2,4‐dichlorobenzyl)‐1H‐indazole‐3‐carboxylic acid), a derivative of indazole‐3‐carboxylic acid, which is composed of indazole carboxylic acid and dichlorobenzene, is known as an antitumor agent that inhibits hexokinase actively produced in cancer cells [31]. Previous studies reported two classes of compounds as modulators for *B. pertussis*: the inorganic ions, SO_4_ and ClO_4_, and organic acids, including nicotinic acid, benzoic acid, and their analogs [27, 54, 55, 56]. While the mode of action by which the inorganic ions inactivate the BvgAS system remains unknown, the common structures of organic acids related to modulator functions were investigated [27]. According to the findings obtained, a planer ring structure, such as a benzene or pyridine ring with a carboxyl group, may be required for the modulating activity: 6‐chloronicotinic acid and 4‐nitrobenzoic acid are representative modulators with these common structures [27]. Lonidamine also has two ring structures, indazole carboxylic acid, and dichlorobenzene, which may meet the above requirements for BvgAS modulators. In the present study, expecting that the potent modulator lonidamine might help us understand the mode of interaction between modulators and BvgS, we also performed a docking simulation and confirmed the importance of indazole carboxylic acid and dichlorobenzene. The modification of the carboxy group bonded to indazole or the exclusion of dichlorobenzene impaired the modulating activity of lonidamine. The minimum effective concentration of lonidamine was estimated to be 50 nM, whereas 6‐chloronicotinic acid and 4‐nitrobenzoic acid exhibited modulating activities at 0.1 and 1 mM, respectively [27]: Lonidamine was 2000‐ to 20,000‐fold more effective than 6‐chloronicotinic acid and 4‐nitrobenzoic acid, which may be because lonidamine comprises two planer rings. The docking simulation consistently showed that lonidamine bound to the VFT2 domain of BvgS via indazole carboxylic acid and dichlorobenzene moieties. Bacteria expressing the mutant VFT2 domain that carries the substitutions of all amino acid residues predicted to contribute to the interaction with lonidamine were less sensitive than the wild‐type strain, validating the simulation results. On the other hand, bacterial mutants still responded to lonidamine at higher concentrations. These results imply that the docking simulation does not provide the entire mechanism of the interaction between lonidamine and the VFT2 domain. Therefore, to obtain a more detailed understanding of the interaction mode, we are now trying to elucidate the actual structure of the VFT2 domain with lonidamine. The periplasmic domain of BvgS shows a dimeric arrangement, housing two sets of VFT1 and VFT2. Previous studies suggested that changes in the relative positions in intra‐ and inter‐protomer interfaces determine the on and off states of the kinase activity of BvgS [26, 51, 57]. Lonidamine may serve as a useful probe to understand how modulators affect the structures of the periplasmic domain of BvgS upon binding to VFT2.

Previous studies indicated that classical *Bordetella* species in the Bvg^–^ phase were unable to colonize and infect host animals [1, 23, 24, 29, 49], supporting the idea that an effective modulator to turn bacteria to the Bvg^–^ phase may be useful for controlling *Bordetella* infection. Lonidamine, which affected the bacteria at nanomolar levels, was expected to be a promising candidate for such modulators. Indeed, the treatment of *B. pertussis* with lonidamine before and after infection reduced bacterial adherence to cultured cells. However, in mouse infection experiments, lonidamine exacerbated bacterial colonization in the nasal septum at a later period of infection. There are several possible reasons for these unexpected results. The bacterial phenotypes may differ between the Bvg^–^ phase‐locked mutant and modulator‐induced Bvg^–^ phase bacteria. The previous studies showing the non‐infectivity of the Bvg^–^ phase bacteria were conducted mainly using the Bvg^–^ phase‐locked mutant, which strictly expresses *vrg*s and does not express *vag*s in vitro [23, 24, 29, 49]. However, recent studies have shown that some *vrg*s are expressed in *B. bronchiseptica* during infection [34, 39], and two‐component systems other than BvgAS, such as RisAS, RisAK, and PlrRS, are involved in the gene expression of *Bordetella* species colonizing respiratory tracts [8, 22, 58, 59]. These findings imply that the pathogenicity of *Bordetella* species cannot be simply explained by the Bvg phases, and their infection processes are more complex than previously considered. Therefore, lonidamine‐modulated bacteria may still be infective in vivo. Furthermore, lonidamine may affect other biological functions of bacteria or host animals. For example, we cannot exclude the possibility that lonidamine affects the signaling cascades of other two‐component systems involved in the establishment of infection. To examine this possibility, we are now analyzing gene expression profiles in *Bordetella* species treated with lonidamine. In addition, the effects of lonidamine on host animals cannot be ignored. Lonidamine has been reported to affect mitochondrial function and disturb the energy metabolism of eukaryotic cells [60], leading to cell apoptosis. Another study identified lonidamine as a broad‐spectrum inflammasome inhibitor [61]. These undesired effects of lonidamine may contribute to the exacerbation of bacterial colonization.

This study demonstrated that lonidamine functions as an effective modulator, which converts classical *Bordetella* species to the avirulent Bvg^–^ phase at nanomolar levels. This novel modulator may be a valuable tool for elucidating the mechanisms by which modulators inactivate the BvgAS system and may also be a reference compound in the search for natural ligands for BvgS. In addition, the use of a modulator to control *Bordetella* infection is worthy of consideration because the resurgence of pertussis and the emergence of macrolide‐resistant *B. pertussis* pose a social issue that must be addressed. We will make continuous efforts to identify more efficient modulators by using lonidamine as a lead compound. For this purpose, the bacterial reporter systems with different Bvg phase‐specific promoters established in the present study will be of great use.

## Disclosure

The authors have nothing to report.

## Ethics Statement

All animal experiments were approved by the Animal Care and Use Committee of the Research Institute for Microbial Disease, Osaka University, and were performed according to the Regulations on Animal Experiments at Osaka University.

## Supporting information

Supporting information.

Supporting information.

Supporting information.

Supporting information.

Supporting information.

## Data Availability

Data are available on request from the authors.
