# Supplementary material for: Lonidamine, a Novel Modulator for the BvgAS System of Bordetella Species
Source: Microbiol Immunol. 2024 Dec 15;69(3):133–47. doi: 10.1111/1348-0421.13193 (PMC11873758; doi:10.1111/1348-0421.13193)
Supplement: Supplementary file 1 — Supporting information. [file MIM-69-133-s003.docx]

**Figure. S1.** The growth of *B. pertussis* Tohama in the presence of candidate compounds.

Bacteria were incubated in SS medium containing each compound at the indicated concentrations (red), 1.5% DMSO (Mock, black) or 50 mM MgSO_4_ (blue). Values represent the mean ± SD (n=3). Light red lines indicate cultures showing reduced or inhibited bacterial growth compared to the MgSO_4_-treated culture. Numbered compounds are fludarabine (#3), lonidamine (#5), dydrogesterone (#6), and otilonium bromide (#8).

**Figure S2.** SDS-PAGE and immunoblotting of recombinant VFT proteins and *B. pertussis* cell lysates.

Recombinant VFT1 (36.5 kDa), VFT2 (31.6 kDa), and VFT1+2 (64.8 kDa) proteins at 4 µg/lane (a, left panel) and 10 ng/lane (a, right panel) and cell lysates of the *bvgS* mutants of *B. pertussis* (Bvg^+^, Bvg^–^, F375A, R380A, T462A+S465A, and quadA; BvgS: 135 kDa) at 3 µg/lane (b) were subjected to 10% SDS-PAGE. After electrophoresis, samples were stained with Coomassie Brilliant Blue R-250 (a, left panel) or subjected to immunoblotting with anti-VFT1+2 serum (a, right panel, and b). FtsZ (41.4 kDa) was simultaneously detected as the internal control to confirm the loading amounts. The arrow indicates the position of BvgS. Note that BvgS was equally detected between wild-type and mutant strains.

**Figure S3.** Effects of lonidamine on Bvg states of *B. pertussis* in the infection experiments.

(a) The ratio of GFP-positive bacteria adhering to A549 cells to mCherry-positive bacteria adhering to A549 cells. Microscopic images of A549 cells infected with mCherry Thm/P*_fhaB_*-*gfp* (mC/P*_fhaB_*-*gfp*) or /P*_vrgX_*-*gfp* (mC/P*_vrgX_*-*gfp*) were captured from at least 8 independent fields and the number of adhering bacteria was counted. A plot symbol represents a single microscopic field. (b) Bioluminescence images of mice infected with P*_vrgX_*-Akaluc Thm. Bacteria were precultured in SS medium and intranasally inoculated into anesthetized mice at 1 × 10^7^ CFU/25 µl/mouse (day 0). Bacteria-infected mice were intranasally injected with (“Lon”) or without (“mock”) 10 mg/kg body weight of lonidamine every day from day 1. On days 1, 4, and 8 of infection, mice were intraperitoneally injected with akalumine-HCl, and bioluminescence images were acquired. On day 1, images were acquired 7 hr before the lonidamine injection. In the mock control, 4.6% DMSO was applied. (c) Quantitative data on luminescence levels in the nasal septum estimated by Living Image 4.7 software. Values represent the mean ± SD (a and n=5 for c). The significance of differences was analyzed by a two-way ANOVA with Šídák’s multiple comparisons test (a) or an unpaired *t*-test on each row with Holm-Šídák’s multiple comparisons test (c).
