# Supplementary figures and images for: Lonidamine, a Novel Modulator for the BvgAS System of Bordetella Species
Source: Microbiol Immunol. 2024 Dec 15;69(3):133–47. doi: 10.1111/1348-0421.13193 (PMC11873758; doi:10.1111/1348-0421.13193)

Figure S1

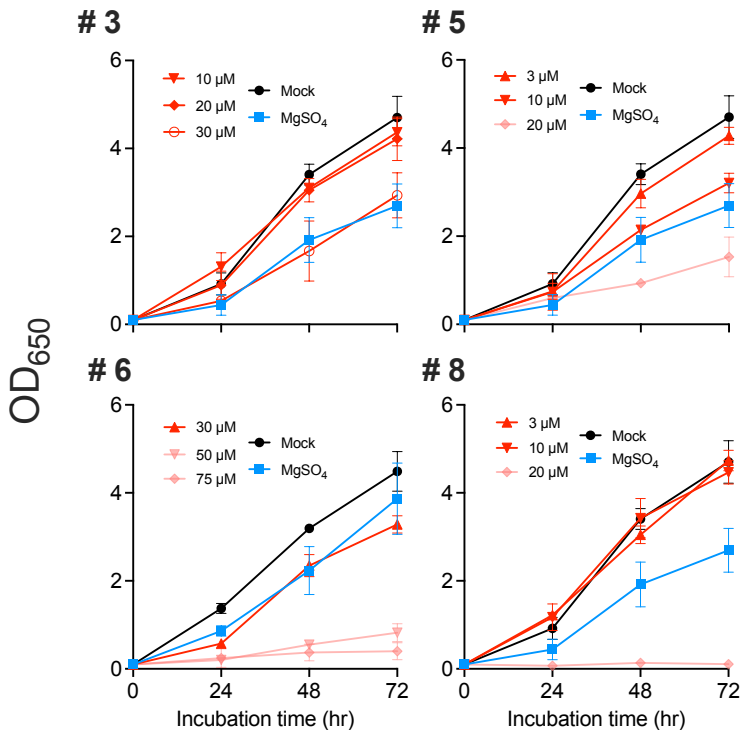

Figure S2

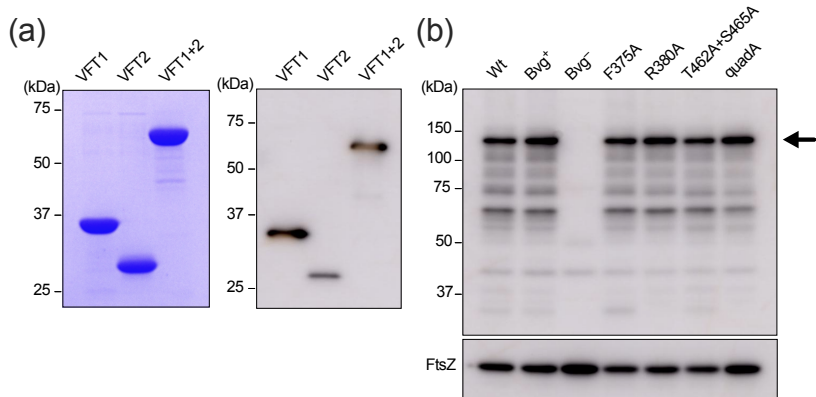

# Figure S3

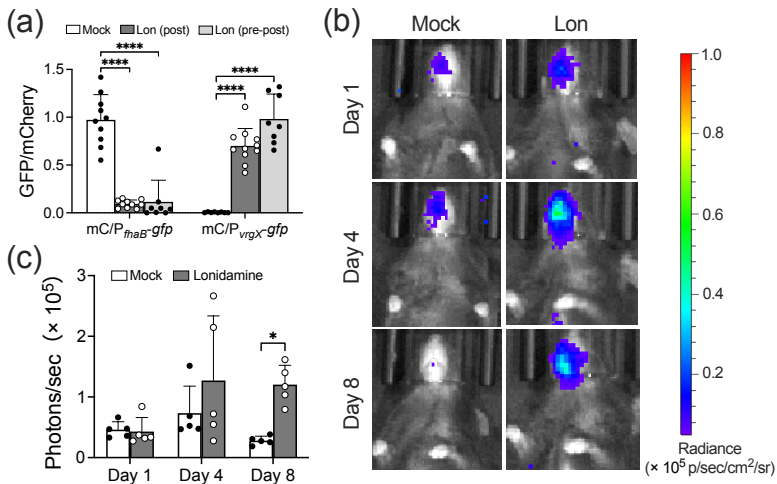

Supplement: Supplementary file 2 — Supporting information. [file MIM-69-133-s002.pdf]
