# Supplementary material for: Lonidamine, a Novel Modulator for the BvgAS System of Bordetella Species
Source: Microbiol Immunol. 2024 Dec 15;69(3):133–47. doi: 10.1111/1348-0421.13193 (PMC11873758; doi:10.1111/1348-0421.13193)
Supplement: Supplementary file 3 — Supporting information. [file MIM-69-133-s005.pdf]

Table S1. Strains used in the present study.

Strains	Description	Source or reference
B. pertussis		
Tohama	Vaccine strain	(1)
18323	Type strain	(2)
BP140	Clinical strain isolated from a pertussis patient	K. Kamachi
BP142	Clinical strain isolated from a pertussis patient	K. Kamachi
Bvg^+ locked Thm	Tohama strain with an amino acid substitution in BvgS from Arg_{570} to His_{570}	This study
Bvg^- locked Thm	Tohama strain with an amino acid deletion in BvgS from Ile_{542} to Val_{1020}	This study
BvgS F375A	Tohama strain with an amino acid substitution in BvgS from Phe_{375} to Ala_{375}	This study
BvgS R380A	Tohama strain with an amino acid substitution in BvgS from Arg_{380} to Ala_{380}	This study
BvgS T462A+S465A	Tohama derivative, producing BvgS, in which Thr and Ser were replaced with Ala at amino acid positions 462 and 465, respectively	This study
BvgS quadA	Tohama derivative, producing BvgS, in which Phe, Arg, Thr, and Ser were replaced with Ala at amino acid positions 375, 380, 462, and 465, respectively	This study
P_{tac}-Akaluc Thm	Tohama derivative, *tac* promoter-driven Akaluc reporter strain	This study
P_{vrgX}-Akaluc Thm	Tohama derivative, *vrgX* promoter-driven Akaluc reporter strain	This study
mCherry Thm	Tohama derivative, *tac* promoter-driven mCherry2 reporter strain	This study
Thm/P_{tac}-*gfp*	Tohama derivative carrying pBBR1MCS5-P_{tac}-*gfp*	This study
Thm/P_{fhaB}-*gfp*	Tohama derivative carrying pBBR1MCS5-P_{fhaB}-*gfp*	This study
Thm/P_{cya}-*gfp*	Tohama derivative carrying pBBR1MCS5-P_{cya}-*gfp*	This study
Thm/P_{dnt}-*gfp*	Tohama derivative carrying pBBR1MCS5-P_{dnt}-*gfp*	This study
Thm/P_{prn}-*gfp*	Tohama derivative carrying pBBR1MCS5-P_{prn}-*gfp*	This study
Thm/P_{ptx}-*gfp*	Tohama derivative carrying pBBR1MCS5-P_{ptx}-*gfp*	This study
Thm/P_{vag8}-*gfp*	Tohama derivative carrying pBBR1MCS5-P_{vag8}-*gfp*	This study
Thm/P_{vrgX}-*gfp*	Tohama derivative carrying pBBR1MCS5-P_{vrgX}-*gfp*	This study
Thm/P_{vrg6}-*gfp*	Tohama derivative carrying pBBR1MCS5-P_{vrg6}-*gfp*	This study
Thm/P_{vrg73}-*gfp*	Tohama derivative carrying pBBR1MCS5-P_{vrg73}-*gfp*	This study
Thm/P_{bp1618}-*gfp*	Tohama derivative carrying pBBR1MCS5-P_{bp1618}-*gfp*	This study
Thm/P_{bp1738}-*gfp*	Tohama derivative carrying pBBR1MCS5-P_{bp1738}-*gfp*	This study
Thm/P_{kpsM}-*gfp*	Tohama derivative carrying pBBR1MCS5-P_{kpsM}-*gfp*	This study
Bvg^+ locked Thm/P_{fhaB}-*gfp*	Tohama Bvg^+ phase-locked derivative carrying pBBR1MCS5-P_{fhaB}-*gfp*	This study
Bvg^+ locked Thm/P_{vrgX}-*gfp*	Tohama Bvg^+ phase-locked derivative carrying pBBR1MCS5-P_{vrgX}-*gfp*	This study
BvgS F375A/P_{fhaB}-*gfp*	Tohama BvgS F375A derivative carrying pBBR1MCS5-P_{fhaB}-*gfp*	This study
BvgS F375A/P_{vrgX}-*gfp*	Tohama BvgS F375A derivative carrying pBBR1MCS5-P_{vrgX}-*gfp*	This study
BvgS R380A/P_{fhaB}-*gfp*	Tohama BvgS F380A derivative carrying pBBR1MCS5-P_{fhaB}-*gfp*	This study
BvgS R380A/P_{vrgX}-*gfp*	Tohama BvgS F380A derivative carrying pBBR1MCS5-P_{vrgX}-*gfp*	This study
BvgS T462A+S465A/P_{fhaB}-*gfp*	Tohama BvgS T462A+S465A derivative carrying pBBR1MCS5-P_{fhaB}-*gfp*	This study
BvgS T462A+S465A/P_{vrgX}-*gfp*	Tohama BvgS T462A+S465A derivative carrying pBBR1MCS5-P_{vrgX}-*gfp*	This study
BvgS quadA/P_{fhaB}-*gfp*	Tohama BvgS F375A+R380A+T462A+S465A derivative carrying pBBR1MCS5-P_{fhaB}-*gfp*	This study
BvgS quadA/P_{vrgX}-*gfp*	Tohama BvgS F375A+R380A+T462A+S465A derivative carrying pBBR1MCS5-P_{vrgX}-*gfp*	This study

mCherry Thm/P_{fhaB}-*gfp*	Tohama mCherry reporter derivative carrying pBBR1MCS5-P_{fhaB}-*gfp*	This study
mCherry Thm/P_{vrgX}-*gfp*	Tohama mCherry reporter derivative carrying pBBR1MCS5-P_{vrgX}-*gfp*	This study
B.parapertussis		
12822	An isolate from a patient with pertussis-like symptoms	A. Abe
B. bronchiseptica		
RB50	An isolate from a rabbit	P. A. Cotter
E. coli		
HB101	K-12 cloning strain	Laboratory collection
DH5α	K-12 cloning strain	Laboratory collection
BL21(DE3)	K-12 cloning strain	Laboratory collection
DH5α λpir	K-12 cloning strain for a plasmid with a R6K origin	Laboratory collection
S17-1 λpir	K-12 cloning strain for a plasmid with a R6K origin	Laboratory collection
BL21/ pColdII-GB1-VFT1	BL21 (DE3) derivative carrying pColdII-GB1-VFT1	This study
BL21/ pColdII-VFT2	BL21 (DE3) derivative carrying pColdII-VFT2	This study
BL21/ pColdII-GB1-VFT1+2	BL21 (DE3) derivative carrying pColdII-GB1-VFT1+2	This study

References

1. KASUGA T, NAKASE Y, UKISHIMA K, TAKATSU K. 1954. Studies on Haemophilus pertussis. V. Relation between the phase of bacilli and the progress of the whooping-cough. Kitasato Arch Exp Med 27:57–62.

2. Park J, Zhang Y, Buboltz AM, Zhang X, Schuster SC, Ahuja U, Liu M, Miller JF, Sebaihia M, Bentley SD, Parkhill J, Harvill ET. 2012. Comparative genomics of the classical *Bordetella* subspecies: the evolution and exchange of virulence-associated diversity amongst closely related pathogens. BMC Genomics 13:545.
