# Supplementary material for: Lonidamine, a Novel Modulator for the BvgAS System of Bordetella Species
Source: Microbiol Immunol. 2024 Dec 15;69(3):133–47. doi: 10.1111/1348-0421.13193 (PMC11873758; doi:10.1111/1348-0421.13193)
Supplement: Supplementary file 4 — Supporting information. [file MIM-69-133-s001.pdf]

Table S2. Plasmids used in the present study.

Plasmids	Description	Source or reference
pRK2013	Km^r, RK2-derivative with a ColE1 replicon containing *tra*, a helper plasmid for conjugative transfer	(1)
pABB-CRS2-Gm	Gm^r, a R6K-derived suicide vector	(2)
pABB-CRS2-Gm-*bvgS*$_{R570H}$	*bvgS* mutation (arginine is replaced with histidine at amino acid position 570) cloned into pABB-CRS2-Gm	This study
pABB-CRS2-Gm-Δ*bvgS*$_{542-1020}$	deletion from 542 to 1020 aa of *bvgS* cloned into pABB-CRS2-Gm	This study
pABB-CRS2-Gm-Tohama BvgS$_{full}$	*bvgS* cloned into pABB-CRS2-Gm	This study
pABB-CRS2-Gm-Tohama BvgS$_{F375A}$	*bvgS* mutation (phenylalanine is replaced with alanine at amino acid position 375) cloned into pABB-CRS2-Gm	This study
pABB-CRS2-Gm-Tohama BvgS$_{R380A}$	*bvgS* mutation (arginine is replaced with alanine at amino acid position 380) cloned into pABB-CRS2-Gm	This study
pABB-CRS2-Gm-Tohama BvgS$_{T462A+S465A}$	*bvgS* mutation (threonine and serine are replaced with alanine at amino acid positions 462 and 465) cloned into pABB-CRS2-Gm	This study
pABB-CRS2-Gm-Tohama BvgS$_{F375A+T462A+S465A}$	*bvgS* mutation (phenylalanine, threonine, and serine are replaced with alanine at amino acid positions 375, 462, and 465) cloned into pABB-CRS2-Gm	This study
pABB-CRS2-Gm-Tohama BvgS$_{F375A+R380A+T462A+S465A}$	*bvgS* mutation (phenylalanine, arginine, threonine, and serine are replaced with alanine at amino acid positions 375, 380, 462, and 465) cloned into pABB-CRS2-Gm	This study
pEX_K4J2_Akaluc^{opt}	Akaluc optimized codon usage against *B. bronchiseptica*	Eurofins Genomics
pBBR1MCS5-P$_{tac}$-*Akaluc opt*-T$_{trpA}$	pBBR1MCS5 carrying the *tac* promoter, *Akaluc opt*, and *trpA* terminator	This study
pABB-CRS2-Gm-Tohama-BP3747-BP3748	pABB-CRS2-Gm carrying the BP3747–BP3748 intergenic region	This study
pBBR1MCS5-BRP1340-Akaluc opt	pBBR1MCS5 carrying the *vrgX* promoter, *Akaluc opt*, and *trpA* terminator	This study
pABB-CRS2-Gm-Tohama-BP3747-BP3748-P$_{tac}$-*Akaluc opt*	pABB-CRS2-Gm-Tohama-BP3747-BP3748 carrying the *tac* promoter, *Akaluc opt*, and *trpA* terminator	This study
pABB-CRS2-Gm-Tohama-BP3747-BP3748-P$_{vrgX}$-*Akaluc opt*	pABB-CRS2-Gm-Tohama-BP3747-BP3748 carrying the *vrgX* promoter, *Akaluc opt*, and *trpA* terminator	This study
pBBR1MCS5-TpR-P$_{tac}$-mCherry2-T$_{trpA}$	pBBR1MCS5 carrying Tp^r, the *tac* promoter, and *mCherry2*	Laboratory collection
pABB-CRS2-Gm-Tohama-BP3747-BP3748-P$_{tac}$-mCherry2	pABB-CRS2-Gm-Tohama-BP3747-BP3748 carrying the *tac* promoter and *mCherry2*	This study
pBBR1MCS5-P$_{tac}$-*gfp*	pBBR1MCS5 carrying the *tac* promoter, *gfp*, and *trpA* terminator	(3)
pBBR1MCS5-P$_{tac}$	pBBR1MCS5 carrying the *tac* promoter	This study
pBBR1MCS5-P$_{fhaB}$-*gfp*	pBBR1MCS5 carrying the *fhaB* promoter, *gfp*, and *trpA* terminator	This study
pBBR1MCS5-P$_{cya}$-*gfp*	pBBR1MCS5 carrying the *cya* promoter, *gfp*, and *trpA* terminator	This study
pBBR1MCS5-P$_{dnt}$-*gfp*	pBBR1MCS5 carrying the *dnt* promoter, *gfp*, and *trpA* terminator	This study
pBBR1MCS5-P$_{prn}$-*gfp*	pBBR1MCS5 carrying the *prn* promoter, *gfp*, and *trpA* terminator	This study
pBBR1MCS5-P$_{ptx}$-*gfp*	pBBR1MCS5 carrying the *ptx* promoter, *gfp*, and *trpA* terminator	This study
pBBR1MCS5-P$_{vag8}$-*gfp*	pBBR1MCS5 carrying the *vag8* promoter, *gfp*, and *trpA* terminator	This study
pBBR1MCS5-P$_{vrgX}$-*gfp*	pBBR1MCS5 carrying the *vrgX* promoter, *gfp*, and *trpA* terminator	This study
pBBR1MCS5-P$_{vrg6}$-*gfp*	pBBR1MCS5 carrying the *vrg6* promoter, *gfp*, and *trpA* terminator	This study
pBBR1MCS5-P$_{vrg73}$-*gfp*	pBBR1MCS5 carrying the *vrg73* promoter, *gfp*, and *trpA* terminator	This study

pBBR1MCS5-P$_{bp1618}$-*gfp*	pBBR1MCS5 carrying the *bp1618* promoter, *gfp*, and *trpA* terminator	This study
pBBR1MCS5-P$_{bp1738}$-*gfp*	pBBR1MCS5 carrying the *bp1738* promoter, *gfp*, and *trpA* terminator	This study
pBBR1MCS5-P$_{kpsM}$-*gfp*	pBBR1MCS5 carrying the *kpsM* promoter, *gfp*, and *trpA* terminator	This study
pColdII	Amp^r, a cold-shock expression vector, with a N-terminal hexa-histidine tag	TaKaRa Bio
pGEV2	Amp^r, the GB1 domain of protein G as a N-terminal tag, C-terminal hexa-histidine tag, expression vector	Addgene
pColdII-GEV2	pCold II carrying the GB1 domain derived from the GEV2 plasmid	This study
pColdII-GB1-VFT1	pCold II-GEV2 carrying the VFT1 domain of *bvgS*	This study
pColdII-VFT2	pCold II carrying the VFT2 domain of *bvgS*	This study
pColdII-GB1-VFT1+2	pCold II-GEV2 carrying the VFT1+2 domain of *bvgS*	This study

References

1. Figurski DH, Helinski DR. 1979. Replication of an origin-containing derivative of plasmid RK2 dependent on a plasmid function provided in *trans*. Proceedings of the National Academy of Sciences 76:1648–1652.

2. Sekiya K, Ohishi M, Ogino T, Tamano K, Sasakawa C, Abe A. 2001. Supermolecular structure of the enteropathogenic *Escherichia coli* type III secretion system and its direct interaction with the EspA-sheath-like structure. Proceedings of the National Academy of Sciences 98:11638–11643.

3. Nishikawa S, Shinzawa N, Nakamura K, Ishigaki K, Abe H, Horiguchi Y. 2016. The *bvg* -repressed gene *brtA*, encoding biofilm-associated surface adhesin, is expressed during host infection by *Bordetella bronchiseptica*. Microbiol Immunol 60:93–105.
