# Supplementary material for: Lonidamine, a Novel Modulator for the BvgAS System of Bordetella Species
Source: Microbiol Immunol. 2024 Dec 15;69(3):133–47. doi: 10.1111/1348-0421.13193 (PMC11873758; doi:10.1111/1348-0421.13193)
Supplement: Supplementary file 5 — Supporting information. [file MIM-69-133-s004.pdf]

Table S3. Primers used in the present study.

Name	Sequence (5' to 3')	Template	Application
BvgS-region1Bp-S+	GATCCGAGCTCTCCCACGCTGCATTACTTCCCCTC	18323 genomic DNA (gDNA)	pABB-CRS2-Gm-*bvgS*$_{\mathrm{R570H}}$
BvgS-region2Bp-AS+	ATTTGTGGAATTCCCCAACCTGGTGCTTACCGTCA		
BB2995-C3mutation-S	GCCAGATCCGCCAGCACAAGCGGGCCGAGCG		
BB2995-C3mutation-AS	CGCTCGGCCCGCTTGTGCTGGCGGATCTGGC		
BvgS-region1Bp-S+	GATCCGAGCTCTCCCACGCTGCATTACTTCCCCTC	18323 gDNA	pABB-CRS2-Gm-Δ*bvgS*$_{\mathrm{542\text{-}1020}}$
BvgS-region2Bp-AS+	ATTTGTGGAATTCCCCAACCTGGTGCTTACCGTCA		
BvgS-region2-S	ATCACCGATTGCAACATGCC		
BvgS-region1-AS	CTCGTTGCGGTAGGCGTA		
BvgS_full-F	CCATCACCAGGCCATCTGGCTGGGGTGCTTTCAGGGCTGATCTTGATGCGGCCGTTGCTC	Tohama gDNA	pABB-CRS2-Gm-Tohama BvgS$_{\mathrm{full}}$
BvgS_full-R	CCCGGGAGAGCTCGGATCCACTAGTCGGTTATGCCCGCCCCGCACCGC		
BD267-F	AACCGACTAGTGGATCCGAGCTCTCCCGG	pABB-CRS2-Gm	
BD264-R	AAGCACCCCAGCCAGATGGCCTGGTGATGGCGGGATCGTT		
F375A-F2	GCGGTCAACAGCGCGCGGGAGTCCTTCCTCAGTTTCAGCC	pABB-CRS2-Gm-Tohama BvgS$_{\mathrm{full}}$	pABB-CRS2-Gm-Tohama BvgS$_{\mathrm{F375A}}$
BD319-R	CAGGGCGCCGGCCATGTCGGCTTCG		
R380A-F2	GCGGAGTCCTTCCTCAGTTTCAGCCGGCCGTATGTGCGC	pABB-CRS2-Gm-Tohama BvgS$_{\mathrm{full}}$	pABB-CRS2-Gm-Tohama BvgS$_{\mathrm{R380A}}$
R380A-R2	CGCGCTGTTGACGAACAGGGCGCCGG		
T462A_S465A-F1	GACGCCGTCGTGCAGGCGCAGATCGCGGCCAGCTATTACGTCAACCGCTACTTCGCC	pABB-CRS2-Gm-Tohama BvgS$_{\mathrm{full}}$	pABB-CRS2-Gm-Tohama BvgS$_{\mathrm{T462A+S465A}}$
T462A_S465A-R1	GGCCTGTCCATTGGCCACCATCAGC		
T462A_S465A-F1	GACGCCGTCGTGCAGGCGCAGATCGCGGCCAGCTATTACGTCAACCGCTACTTCGCC	pABB-CRS2-Gm-Tohama BvgS$_{\mathrm{F375A}}$	pABB-CRS2-Gm-Tohama BvgS$_{\mathrm{F375A+T462A+S465A}}$
T462A_S465A-R1	GGCCTGTCCATTGGCCACCATCAGC		
R380A-F2	GCGGAGTCCTTCCTCAGTTTCAGCCGGCCGTATGTGCGC	pABB-CRS2-Gm-Tohama BvgS$_{\mathrm{F375A+T462A+S465A}}$	pABB-CRS2-Gm-Tohama BvgS$_{\mathrm{F375A+R380A+T462A+S465A}}$
R380A_F375A-R1	CGCGCTGTTGACCGCCAGGGCGC		
Akaluc opt S+	ACAGGAAACAGAATTCATGGAAGACGCGAAGAACATC	pEX_K4J2_Akaluc^{opt}	pBBR1MCS5-P$_{tac}$-Akaluc opt-T$_{trpA}$
Akaluc opt AS+	CCTGAAAATCGAATTCTCACACGGCGATCTTGC		
In-fusion BRP1340-Fw	CGCTCTAGAACTAGTGGATCACGCGGGCGCGAGTGTTGCG	Tohama gDNA	pBBR1MCS5-BRP1340-Akaluc opt
In-fusion BRP1340-Akaluc-Rv	ATGTTCTTCGCGTCTTCCATGGTCGACACTCCTGTTGATG		
Akaluc opt-Fw	ATGGAAGACGCGAAGAACAT	pBBR1MCS5-P$_{tac}$-Akaluc opt-T$_{trpA}$	
Akaluc opt AS +	CCTGAAAATCGAATTCTCACACGGCGATCTTGC		
Up-BP3747-F1	GGAGAGCTCGGATCCACTAGTCGGTTTGGAGCCATCTTGTCCGATTCCG	Tohama gDNA	

Down-BP3748-R1	ACCAGGCCATCTGGCTGGGGTGCTTATGCCTGTAGACAACCTTCCCCTG		pABB-CRS2-Gm-Tohama BP3747-BP3748
BD267-F	AACCGACTAGTGGATCCGAGCTCTCCCGG	pABB-CRS2-Gm	
BD264-R	AAGCACCCCAGCCAGATGGCCTGGTGATGGCGGGATCGTT		
pABB-Akaluc-F1	TTTAGTTTCCAGCGTGCCAGGCCGTCGCTCAAGGCGC	pBBR1MCS5-P_{tac}-Akaluc opt-T_{trpA}	pABB-CRS2-Gm-Tohama BP3747-BP3748-P_{tac}-Akaluc opt
pABB-Akaluc-R1	ACAAAATACTTGTCCGCCACCCCCCCCTCGAGGTCGAC		
Down-BP3748-F1	ACCGTCGACCTCGAGGGGGGGGTGGCGGACAAGTATTTTGTATCTGAAC	pABB-CRS2-Gm-Tohama BP3747-BP3748	
Up-BP3747-R1	TGCGCCTTGAGCGACGGCCTGGCACGCTGGAAACTAAATCG		
pABB-PfhaB-Akaluc-F1	TTTAGTTTCCAGCGTGCCAGGATCCTATACCAAAATAGGAAAAGTCTGAATTCCTGC	pBBR1MCS5-P_{fhaB}-Akaluc opt	pABB-CRS2-Gm-Tohama BP3747-BP3748-P_{fhaB}-Akaluc opt
pABB-PfhaB-Akaluc-R1	ACAAAATACTTGTCCGCCACCCCCCCCTCGAGGTCGA		
Inverse-BP3748-F1	GTGGCGGACAAGTATTTTGTATCTGAACG	pABB-CRS2-Gm-Tohama BP3747-BP3748	
Inverse-BP3747-R1	CCTGGCACGCTGGAAACTAAATCG		
pABB-BRP1340-Akaluc-F1	TTTAGTTTCCAGCGTGCCAGGCACGCGGGCGCGAGTGTTGC	pBBR1MCS5-BRP1340-Akaluc opt	pABB-CRS2-Gm-Tohama BP3747-BP3748-P_{vrgX}-Akaluc opt
pABB-BRP1340-Akaluc-R1	ACAAAATACTTGTCCGCCACCCCCCCCTCGAGGTCGACGGT		
Inverse-BP3748-F1	GTGGCGGACAAGTATTTTGTATCTGAACG	pABB-CRS2-Gm-Tohama BP3747-BP3748	
Inverse-BP3747-R1	CCTGGCACGCTGGAAACTAAATCG		
pABB-mCherry-F1	TTTAGTTTCCAGCGTGCCAGGTGCGCCGACATCATAACGG	pBBR1MCS5-TpR-P_{tac}-mCherry2-T_{trpA}	pABB-CRS2-Gm-Tohama BP3747-BP3748-P_{tac}-mCherry
pABB-mCherry-R1	ACAAAATACTTGTCCGCCACCGGTTCTCCTACATGTTCGCCT		
Inverse-BP3748-F1	GTGGCGGACAAGTATTTTGTATCTGAACG	pABB-CRS2-Gm-Tohama BP3747-BP3748	
Inverse-BP3747-R1	CCTGGCACGCTGGAAACTAAATCG		
Ki31	CAACCGTTGGCATGGCCAAGT	DNA from a colony	Sequencing of *mCherry2* in mCherry Thm
pABB-check-R1	ATGCAAGACAGGCCGCTACG		
In-fusion PfhaB-Fw	CGCTCTAGAACTAGTGGATCATCCTATACCAAAATAGGAAAAG	18323 gDNA	pBBR1MCS5-P_{fhaB}-*gfp*
In-fusion PfhaB-Rv	GATTTAAAGATCCCCGGGAATTCCAGTGCCATAGTCTGGATAAACC		
In-fusion PfhaB-gfp-Fw	GGTTTATCCAGACTATGGCACTGGAATTCCCGGGGATCTTTAAATC	pBBR1MCS5-P_{tac}-*gfp*	
In-fusion gfp-Rv	TAGCTCCTGAAAATCGAATTCTATTTGTATAGTTCATCCA		
In-fusion Pcya-Fw	CGCTCTAGAACTAGTGGATCTGCGAGCAGATGCACCGGCC	Tohama gDNA	pBBR1MCS5-P_{cya}-*gfp*
In-fusion Pcya-Rv	AGTTCTTCTCCTTTACTCATGTGGATCTGTCGATAAGTAG		
In-fusion Pcya-gfp-Fw	CTACTTATCGACAGATCCACATGAGTAAAGGAGAAGAACT	pBBR1MCS5-P_{tac}-*gfp*	
In-fusion gfp-Rv	TAGCTCCTGAAAATCGAATTCTATTTGTATAGTTCATCCA		

In-fusion Pdnt-Fw	CGCTCTAGAACTAGTGGATCGTCCGGCCTGGCCTGGCGAT	Tohama gDNA	pBBR1MCS5-P_{dnt}-*gfp*
In-fusion Pdnt-Rv	AGTTCTTCTCCTTTACTCATGTTTGTTTGCCCCTGACCGG		
In-fusion Pdnt-gfp-Fw	CCGGTCAGGGGCAAACAAACATGAGTAAAGGAGAAGAACT	pBBR1MCS5-P_{tac}-*gfp*	
In-fusion gfp-Rv	TAGCTCCTGAAAATCGAATTCTATTTGTATAGTTCATCCA		
In-fusion Pprn-Fw	CGCTCTAGAACTAGTGGATCGGGGACAGGCACCCTGGCCT	Tohama gDNA	pBBR1MCS5-P_{prn}-*gfp*
In-fusion Pprn-Rv	AGTTCTTCTCCTTTACTCATTGGATGCCAGGTGGAGAGCA		
In-fusion Pprn-GFP-Fw	TGCTCTCCACCTGGCATCCAATGAGTAAAGGAGAAGAACT	pBBR1MCS5-P_{tac}-*gfp*	
In-fusion gfp-Rv	TAGCTCCTGAAAATCGAATTCTATTTGTATAGTTCATCCA		
In-fusion Pptx-Fw	CCGCTCTAGAACTAGTGGATCCAGCGCTGGGCCGGCGTCG	Tohama gDNA	pBBR1MCS5-P_{ptx}-*gfp*
In-fusion Pptx-Rv	AGTTCTTCTCCTTTACTCATCCCGTCTTCCCCTCTGCGTT		
In-fusion Pptx-gfp-Fw	AACGCAGAGGGGAAGACGGGATGAGTAAAGGAGAAGAACT	pBBR1MCS5-P_{tac}-*gfp*	
In-fusion gfp-Rv	TAGCTCCTGAAAATCGAATTCTATTTGTATAGTTCATCCA		
In-fusion Pvag8-Fw	AGTTCTTCTCCTTTACTCATCTCAACACCTCTTGGCTAGA	Tohama gDNA	pBBR1MCS5-P_{vag8}-*gfp*
In-fusion Pvag8-Rv	CGCTCTAGAACTAGTGGATCGTCTTAATTATTACCGCTGG		
In-fusion Pvag8-gfp-Fw	TCTAGCCAAGAGGTGTTGAGATGAGTAAAGGAGAAGAACT	pBBR1MCS5-P_{tac}-*gfp*	
In-fusion gfp-Rv	TAGCTCCTGAAAATCGAATTCTATTTGTATAGTTCATCCA		
In-fusion *vrgX*-Fw	CGCTCTAGAACTAGTGGATCACGCGGGCGCGAGTGTTGCG	Tohama gDNA	pBBR1MCS5-P_{vrgX}-*gfp*
In-fusion *vrgX*-Rv	AGTTCTTCTCCTTTACTCATGGTCGACACTCCTGTTGATG		
In-fusion *vrgX*-gfp-Fw	CATCAACAGGAGTGTCGACCATGAGTAAAGGAGAAGAACT	pBBR1MCS5-P_{tac}-*gfp*	
In-fusion gfp-Rv	TAGCTCCTGAAAATCGAATTCTATTTGTATAGTTCATCCA		
In-fusion Pvrg6-Fw	CGCTCTAGAACTAGTGGATCATACTGCCACACATGACACC	Tohama gDNA	pBBR1MCS5-P_{vrg6}-*gfp*
In-fusion Pvrg6-Rv	AGTTCTTCTCCTTTACTCATTTTTCACCTGCCTTGCTGCG		
In-fusion Pvrg6-gfp-Fw	CGCAGCAAGGCAGGTGAAAAATGAGTAAAGGAGAAGAACT	pBBR1MCS5-P_{tac}-*gfp*	
In-fusion gfp-Rv	TAGCTCCTGAAAATCGAATTCTATTTGTATAGTTCATCCA		
In-fusion Pvrg73-Fw	CCGCTCTAGAACTAGTGGATCTACCGCAGCGACTACATCA	Tohama gDNA	pBBR1MCS5-P_{vrg73}-*gfp*
In-fusion Pvrg73-Rv	AGTTCTTCTCCTTTACTCATGAAGGGCTCCTTGCTTGTTG		
In-fusion Pvrg73-gfp-Fw	CAACAAGCAAGGAGCCCTTCATGAGTAAAGGAGAAGAACT	pBBR1MCS5-P_{tac}-*gfp*	
In-fusion gfp-Rv	TAGCTCCTGAAAATCGAATTCTATTTGTATAGTTCATCCA		
In-fusion Pbp1618-Fw	CGCTCTAGAACTAGTGGATCGCCATGCCAGCCCCGAGCCC	Tohama gDNA	pBBR1MCS5-P_{bp1618}-*gfp*
In-fusion Pbp1618-Rv	AGTTCTTCTCCTTTACTCATGCGGCACGAACCTATTGTTG		

In-fusion Pbp1618-gfp-Fw	CAACAATAGGTTCGTGCCGCATGAGTAAAGGAGAAGAACT	pBBR1MCS5-P_{tac}-*gfp*	
In-fusion gfp-Rv	TAGCTCCTGAAAATCGAATTCTATTTGTATAGTTCATCCA		
In-fusion Pbp1738-Fw	CCGCTCTAGAACTAGTGGATCAAGCGTGGTTACCTGAGCC	Tohama gDNA	pBBR1MCS5-P_{bp1738}-*gfp*
In-fusion Pbp1738-Rv	AGTTCTTCTCCTTTACTCATGGTTGTCCTCTTCTAGGTCG		
In-fusion Pbp1738-gfp-Fw	CGACCTAGAAGAGGACAACCATGAGTAAAGGAGAAGAACT	pBBR1MCS5-P_{tac}-*gfp*	
In-fusion gfp-Rv	TAGCTCCTGAAAATCGAATTCTATTTGTATAGTTCATCCA		
In-fusion PkpsM-Fw	CGCTCTAGAACTAGTGGATCGGCGGCCTTGCAAGTGTGCC	Tohama gDNA	pBBR1MCS5-P_{kpsM}-*gfp*
In-fusion PkpsM-Rv	AGTTCTTCTCCTTTACTCATACCTGGGCTCCCGATGCCTG		
In-fusion PkpsM-gfp-Fw	CAGGCATCGGGAGCCCAGGTATGAGTAAAGGAGAAGAACT	pBBR1MCS5-P_{tac}-*gfp*	
In-fusion gfp-Rv	TAGCTCCTGAAAATCGAATTCTATTTGTATAGTTCATCCA		
GEV2-insert-F1	ACACCATGAATCACAAAGTGCATATGCAGTACAAGCTTGCTCTGAACGG	pGEV2	pColdII-GEV2
GEV2-insert-R1	TGCTTTTAAGCAGAGATTACTCAGTGGTGGTGGTGGTGGTGC		
pCold-inverse-F1	GTAATCTCTGCTTAAAAGCACAGAATCTAAGATCCCTGC	pColdII	
pCold-inverse-R1	CACTTTGTGATTCATGGTGTATTACCTCTTAATAATTAAGTGTGCC		
VFT1-F1	TAGGATCCGCGCAGGCAAGCCAG	pABB-CRS2-Gm-Tohama BvgS$_{full}$	pColdII-GB1-VFT1
VFT1-R1	ATCTCGAGCGAAATGCTGCTGCC		
VFT2-F1	ATGGATCCCTCGATTTCGCGCACC	pABB-CRS2-Gm-Tohama BvgS$_{full}$	pColdII-VFT2
VFT2-R1	ATAAGCTTGTAGATCTCGTTGCGGTAGGC		
VFT1-F1	TAGGATCCGCGCAGGCAAGCCAG	pABB-CRS2-Gm-Tohama BvgS$_{full}$	pColdII-GB1-VFT1+2
VFT1+2-R1	TACTCGAGGTAGATCTCGTTGCGGTAGGC		
recA-Fw	CCAATGTGGTCGACAAGTCC	Tohama complementary DNA (cDNA)	Real-time PCR targeting *recA*
recA-Rv	ATGGCCATTTCCTTGTGCTC		
qfhaB-Fw	CGTTGAACTGCTGGAACTTGTT	*Bordetella* cDNA	Real-time PCR targeting *fhaB*
qfhaB-Rv	AAGGTTCCCGTTGTCAATATCG		
qprn-Fw	ACCAGTCCATCGTCAAGACC	*B. pertussis* cDNA	Real-time PCR targeting *prn*
qprn-Rv	CGCTTACCTTGATGGTGGTT		
qdnt-Fw	ATGACGCGGCTTATGTATCC	*Bordetella* cDNA	Real-time PCR targeting *dnt*
qdnt-Rv	CTGCTCCAGCCTTCTGAATC		
qfim2-Fw	AGATTGGAAATCCGTACCTG	*B. pertussis* cDNA	Real-time PCR targeting *fim2*
qfim2-Rv	AGATGGTCTATGCCACCAAT		
qbscN-Fw	TGTAAGCAAACCGTCCAGTA	*Bordetella* cDNA	Real-time PCR targeting *bscN*
qbscN-Rv	CCACAAGTTCTATCCGGTCT		
qcyaA-Fw	TGGCACTGAGCAGAACAATC	*Bordetella* cDNA	Real-time PCR targeting *cyaA*
qcyaA-Rv	CTGCTGGCCAATGTATTCCT		
qptxA-Fw	CACACCGGCGCATTCC	*B. pertussis* cDNA	Real-time PCR targeting *ptxA*
qptxA-Rv	TTGTGATAGACCCGCGTTACC		

qvag8-Fw	GCGTATGTCTCCTACCTGAATG	*Bordetella* cDNA	Real-time PCR targeting *vag8*
qvag8-Rv	GCCGAAAACGGTTGTACTTG		
qBRP1340 -Fw	ATGAGCAAAACCTTGATTCTGG	*B. pertussis* cDNA	Real-time PCR targeting *vrgX*
qBRP1340 -Rv	CGTCTTCGTTCTTGTTGCAG		
qvrg73-Fw	AGCTACTTCAACCTGTTCGC	*B. pertussis* cDNA	Real-time PCR targeting *vrg73*
qvrg73-Rv	TCGAGCATGGGATAGTCGTTG		
qfim3-Fw	AGATTGGAAATCCGTACCTG	*B. pertussis* cDNA	Real-time PCR targeting *fim3*
qfim3-Rv	AGATGGTCTATGCCACCAAT		
qprn-Fw for Bpp12822, RB50	ATGCCACCTATATCGCCAAC	12822 and RB50 cDNA	Real-time PCR targeting *prn*
qprn-Rv for Bpp12822, RB50	CGCCACCTTGAAGTCATTTT		
qfim2-Fw for Bpp12822, RB50	TGCCCAAGATATCCAAGAGC	12822 and RB50 cDNA	Real-time PCR targeting *fim2*
qfim2-Rv for Bpp12822, RB50	CGAAGTACGCTTTCACACCA		
qflaA-Fw	AAGATTCGGACTACGCGACC	12822 and RB50 cDNA	Real-time PCR targeting of *flaA*
qflaA-Rv	CGTTTTGCGGGACTTGGTTG		
